# Genomic analyses provide insights into peach local adaptation and responses to climate change

**DOI:** 10.1101/gr.261032.120

**Published:** 2021-04

**Authors:** Yong Li, Ke Cao, Nan Li, Gengrui Zhu, Weichao Fang, Changwen Chen, Xinwei Wang, Jian Guo, Qi Wang, Tiyu Ding, Jiao Wang, Liping Guan, Junxiu Wang, Kuozhan Liu, Wenwu Guo, Pere Arús, Sanwen Huang, Zhangjun Fei, Lirong Wang

**Affiliations:** 1 Zhengzhou Fruit Research Institute, Chinese Academy of Agricultural Sciences, Zhengzhou 450009, China;; 2 National Horticulture Germplasm Resources Center, Zhengzhou Fruit Research Institute, Chinese Academy of Agricultural Sciences, Zhengzhou 450009, China;; 3 Key Laboratory of Horticultural Plant Biology (Ministry of Education), College of Horticulture & Forestry Sciences, Huazhong Agricultural University, Wuhan 430000, China;; 4 Agricultural Genome Institute at Shenzhen, Chinese Academy of Agricultural Sciences, Shenzhen 518000, China;; 5 IRTA–Centre de Recerca en Agrigenòmica (CSIC-IRTA-UAB-UB), Barcelona 08193, Spain;; 6 Boyce Thompson Institute for Plant Research, Cornell University, Ithaca, New York 14853, USA;; 7 U.S. Department of Agriculture–Agricultural Research Service, Robert W. Holley Center for Agriculture and Health, Ithaca, New York 14853, USA

## Abstract

The environment has constantly shaped plant genomes, but the genetic bases underlying how plants adapt to environmental influences remain largely unknown. We constructed a high-density genomic variation map of 263 geographically representative peach landraces and wild relatives. A combination of whole-genome selection scans and genome-wide environmental association studies (GWEAS) was performed to reveal the genomic bases of peach adaptation to diverse climates. A total of 2092 selective sweeps that underlie local adaptation to both mild and extreme climates were identified, including 339 sweeps conferring genomic pattern of adaptation to high altitudes. Using genome-wide environmental association studies (GWEAS), a total of 2755 genomic loci strongly associated with 51 specific environmental variables were detected. The molecular mechanism underlying adaptive evolution of high drought, strong UVB, cold hardiness, sugar content, flesh color, and bloom date were revealed. Finally, based on 30 yr of observation, a candidate gene associated with bloom date advance, representing peach responses to global warming, was identified. Collectively, our study provides insights into molecular bases of how environments have shaped peach genomes by natural selection and adds candidate genes for future studies on evolutionary genetics, adaptation to climate changes, and breeding.

Environmental adaptation is fundamental to species survival and conservation of biodiversity, especially under threats of climate change (Blanquart et al. 2013). Unlike animals, which can escape from hostile environments, plants are sessile and have to adapt by shaping and/or fixing genetic variants that are conducive for survival. Generally, climate is the major selective pressure driving adaptive evolution, resulting in different ecotypes within a single species (Fournier-Level et al. 2011; Hancock et al. 2011). However, the mechanisms underlying how climate shapes plant genomes remain largely unclear. Recently, identifying adaptive variants and understanding molecular mechanism of adaptation across a genome have become tractable due to the advances of sequencing technologies. Recent studies have sought to elucidate genetic bases of adaptation through genome-wide identification of selective sweeps as well as loci that associate with climate variables in several species, including *Arabidopsis thaliana* (Fournier-Level et al. 2011), pine (Eckert et al. 2010a,b; Dillon et al. 2013; De La Torre et al. 2019), rice (Qiu et al. 2017), sorghum (Lasky et al. 2015), soybean (Lu et al. 2017, 2020), spruce (Holliday et al. 2010; Yeaman et al. 2016), poplar (Evans et al. 2014; Holliday et al. 2016; Wang et al. 2018; Zhang et al. 2019), and fruit fly (Castellano et al. 2018; Chen et al. 2020). In addition, genomic loci or genes controlling adaptive traits and their adaptive evolution patterns have been revealed through association studies or genetic mapping (Pelgas et al. 2011; Yan et al. 2013; Lu et al. 2017; Navarro et al. 2017; Wang et al. 2018; Shi et al. 2020). However, very few studies have focused on genetic bases of adaptation in domesticated perennial fruit crops. Domesticated crops have adapted to diverse climates during domestication and subsequent spread and show local adaptation through long-term natural selection. Landraces and wild relatives harbor great genetic diversity and an abundance of resistance genes, which provide excellent resources for breeding initiatives. This is especially the case with accessions originating from stressful environments (Bolger et al. 2014). However, a cost of domestication is that many resistance-related genes have been lost (Li et al. 2018; Gao et al. 2019; Wang et al. 2020). In addition, global climate change is driving decreases in productivity and changes of distribution in several crop species (Wheeler and von Braun 2013). Therefore, it is of great importance to identify adaptive genes that can contribute to crop improvement, species survival, and global food security in the face of environmental deterioration.

Peach is an important temperate fruit species, with a global yield of 24.5 million tons in 2018 (FA OSTAT; http://www.fao.org/faostat). It is also an important model system for the Rosaceae family, members of which provide one of the world's main resources of fruits. Peach originated in southwestern China, and its landraces and wild relatives are widespread in both temperate and subtropical regions, as well as in wet and dry climates (Wang et al. 2012). Moreover, peach and its wild relatives can also be found in extremely harsh environments, such as high altitude, severe cold, and high drought regions. On the grounds of wide distributions, peach can be regarded as an excellent material for studying adaptation genetics. Peach has a relatively small genome size (∼227.4 Mb), and genomic analyses have identified a number of loci and genes associated with human selection and agronomically important traits (Falchi et al. 2013; Verde et al. 2013; Cao et al. 2014, 2016, 2019; Li et al. 2019; Zhou et al. 2021), such as fruit size, sugar content, fruit shape, flesh color, etc. However, there have been few studies describing genomic loci associated with environmental adaptation and natural selection.

Our previous studies have revealed the impacts of human selection on peach genomes (Cao et al. 2014, 2019; Li et al. 2019). In this study, we focused on how natural selection shapes the genomes and how peaches have adapted to different environments. We analyzed genomes of a wide collection of 263 peach accessions from a broad range of geographical origins and associated with diverse climates (Fig. 1A), spanning mild and extreme environments, using the resequencing data with an average depth of 5.7× (Supplemental Table S1). We deciphered adaptive patterns across the peach genome by combining the identification of signatures of selective sweeps with genome-wide association studies of environmental variables and adaptive traits. Finally, we also identified a candidate gene associated with peach responses to global warming, based on observations over a 30-yr period.

**Figure 1.**
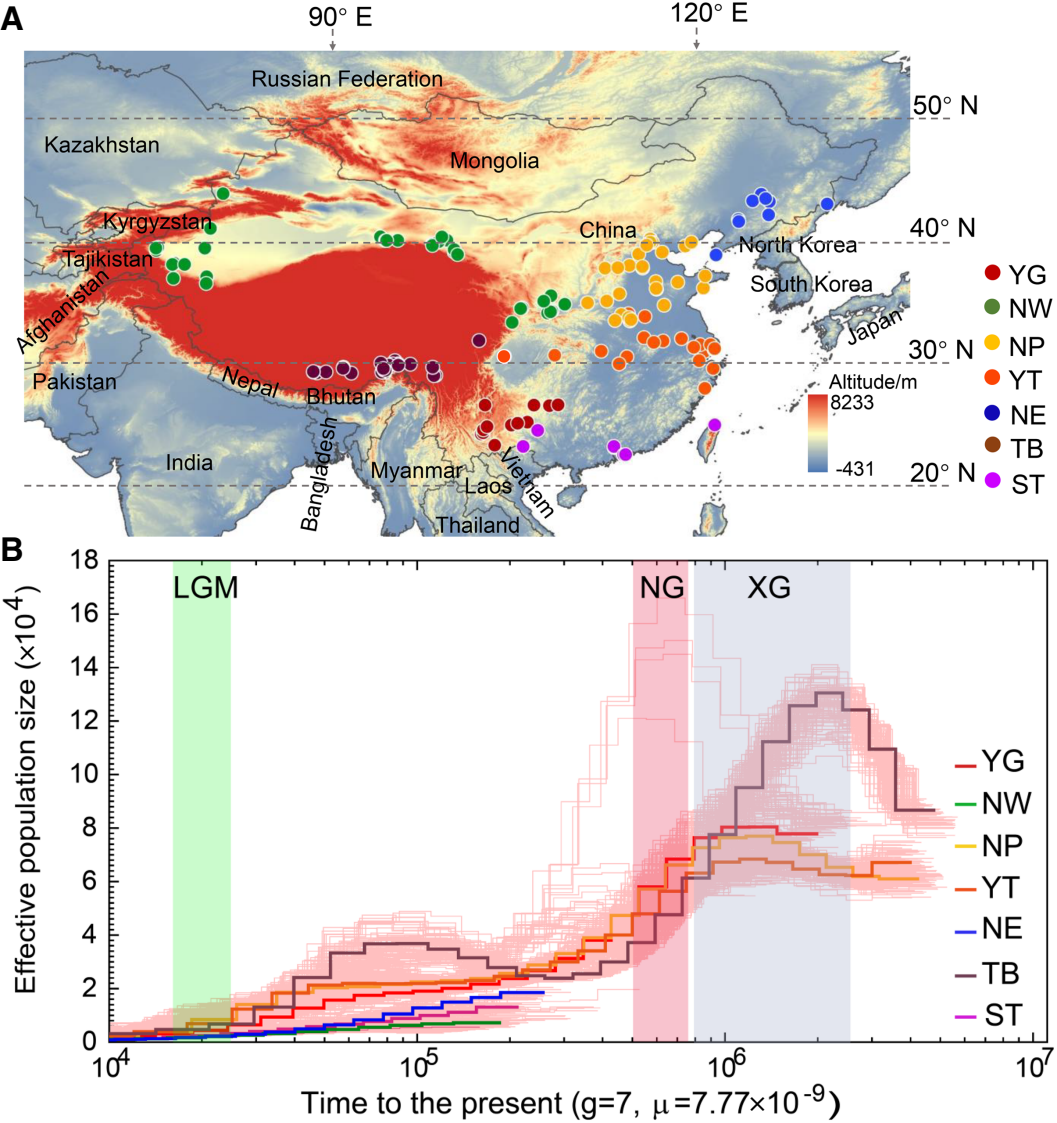
Distribution of the 263 peach accessions and demographic history of the seven ecotypes. (*A*) Geographic distribution of 263 peach accessions used in this study. Each accession is represented by a dot on the world map. Seven ecotypes are highlighted using solid circles with different colors. (*B*) Demographic history of the seven peach groups. Ancestral population size was inferred using the PSMC model. Three periods, the last glacial maximum (LGM, ∼20 KYA), Naynayxungla Glaciation (NG, 0.5∼0.78 MYA), and Xixiabangma Glaciation (XG, 0.8∼0.17 MYA), are shaded in green, red, and blue, respectively.

## Results

### Genomic variation map and population structure

We first constructed a genome variation map for peach using a collection of 263 diverse accessions (Fig. 1A), consisting of 52 wild relatives and 211 landraces (Supplemental Table S1). These accessions were rarely used in commercial production. Based on the climate investigations of origin areas of the 263 accessions, we found that collectively these accessions captured more than 95% of the geographic diversity of the native distribution of peach landraces and wild relatives. Sequencing data of 260 accessions have been reported previously (Li et al. 2019) and three were newly sequenced in this study. In total, 342.7 Gb of paired-end sequences were used, with a median depth of 5.3× and coverage of 91.7% of the reference peach “Lovell” genome (release v2.0) (Supplemental Table S1; Verde et al. 2013). We identified a final set of 4,611,842 high-quality single-nucleotide polymorphisms (SNPs) (Supplemental Fig. S1A), of which 1,931,310 were intronic (∼11.33%) and 848,638 (∼4.98%) were exonic. The accuracy of identified SNPs was found to be ∼95.6%, based on genotyping of 18 randomly selected SNPs in 130 accessions using a Sequenom MassARRAY platform (Supplemental Table S2). In addition, we also identified 1,049,266 small insertions and deletions (indels) (shorter than or equal to 6 bp) and 106,388 large structural variations (SVs; >30 bp) (Supplemental Fig. S1A).

We explored the genetic relationships among 263 accessions using 3,429,878 SNPs with minor allele frequency (MAF) greater than 0.01. Based on the neighbor-joining tree and population structure analyses, the 263 peach accessions could be divided into seven major groups (best *K* = 7) (Supplemental Fig. S2A,B), which were largely congruent with ecotypes classified according to their geographic information (Wang and Zhuang 2001), including YG (Yun-gui plateau), NW (Northwest China), NP (North Plain China), YT (Yangtze River Middle and Backward), NE (Northeast China), TB (Tibet plateau), and ST (South China Subtropical) groups (Supplemental Figs. S1B, S2; Supplemental Table S1). Although the neighbor-joining tree largely supported the division of seven major groups, there were some discrepancies between geographical characterization and phylogenetic clustering (Supplemental Fig. S2D), indicating the shared ancestral variations and historical gene flows among landraces in closely related groups. Moreover, principal component analysis (PCA) and model-based clustering analyses also supported the extensive admixture and possible gene flow among landrace groups (Supplemental Fig. S2E,F). Furthermore, we found small or moderate pairwise genetic differentiation (*F*_ST_) values (0.013 ± 0.0015∼0.097 ± 0.012) between different landrace groups, again consistent with population admixture (Supplemental Fig. S2G,H).

Using the demographic analysis with the pairwise sequential Markovian coalescent (PSMC) model (Supplemental Methods; Li and Durbin 2011), we found a sharp decline of effective population size (*Ne*) during the two largest Pleistocene glaciations: the Xixiabangma glaciation (XG, 1.17–0.8 MYA) and Naynayxungla glaciation (NG, 0.78–0.50 MYA), and a slight decline of *Ne* during the last glacial maximum (LGM, ∼20,000 yr ago) (Fig. 1B). In addition, for the TB group, we found a slight population expansion after the Naynayxungla glaciation at ∼0.3 MYA.

### Selective sweeps related to adaptation to diverse environments

**Figure 2.**
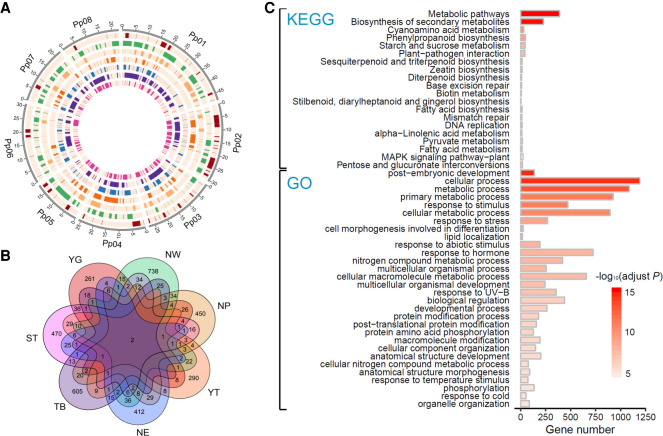
Summary of genes under selection for the seven peach groups. (*A*) Circos plot of the selective sweeps for the seven groups (Supplemental Table S4). The Circos plot in SVG format is available at figshare (https://figshare.com/articles/figure/Genomic_analyses_provide_insights_into_peach_local_adaptation_and_responses_to_climate_change/13158482). The *outer* track represents the eight chromosomes of the peach genome. The seven *inner* tracks depict the distribution of selective sweeps across the peach genome in YG, NW, NP, YT, NE, TB, and ST groups, respectively (from the outside inward). (*B*) Venn diagram showing the number of genes under selection in the seven groups. (*C*) Overrepresented Gene Ontology (GO) terms and Kyoto Encyclopedia of Genes and Genomes (KEGG) pathways in overall selection regions. Only the top 20 and 30 most overrepresented KEGG pathways and GO terms are shown. (YG) Yun-gui plateau, (NW) Northwest China, (NP) North Plain China, (YT) Yangtze River Middle and Backward, (NE) Northeast China, (TB) Tibet plateau, (ST) South China Subtropical.

Peach accessions of each group have adapted locally through long-term selection under local environments (Supplemental Table S3). To identify genomic loci that favor local adaptation for the seven groups, we detected signatures of selective sweeps for each group. This revealed a total of 2092 genomic regions for all seven groups (19.1 Mb, ∼8.4%; 189, 387, 301, 235, 280, 339, and 378 regions for the YG, NW, NP, YT, NE, TB, and ST groups, respectively) (Fig. 2A; Supplemental Table S4), which were termed candidate selection regions (CSRs). Together, these CSRs harbored 3742 genes (∼13.9%), including 396, 966, 635, 403, 573, 743, and 680 genes for the YG, NW, NP, YT, NE, TB, and ST groups, respectively (Fig. 2B). Selections on these genes may underlie the genetic bases of adaptation to biotic and abiotic factors in different climates of peach. We found that few genes were shared among different groups (Fig. 2B), suggesting the unique adaptive patterns for each group and that different climates may shape distinct genomic regions.

We found that genes related to response to different types of stimuli and stress, including temperature, radiation, salt, DNA damage, osmotic, toxin, and biotic stimulus, were overrepresented (adjusted *P* < 0.05), suggesting that stress-related genes have participated in adaptive evolution (Fig. 2C; Supplemental Table S5; Supplemental Fig. S3). For instance, two cation/H^+^ exchanger family genes (*CHX*; *Prupe.6G251600* and *Prupe.6G251700*) and one SALT OVERLY SENSITIVE 3 (*SOS3*; *Prupe.2G188700*) gene showed a high reduction of nucleotide diversity (ROD) in the NW group compared to the other five groups and high *F*_ST_ values between the NW group and the other five groups. Homologs of these genes are involved in salt resistance in *A. thaliana* (Monihan et al. 2016), suggesting their potential contributions to adaptation to saline soils in northwestern China. In addition, we found that genes with the LRR domain, which is considered to be one of the most important domains involved in plant resistance, were also enriched, with 121 of 612 members (∼19.8%) in CSRs. PPR proteins form one of the largest protein families in land plants that are related to environmental responses, with 286 members in the peach genome, of which 79 (∼27.6%) were in CSRs.

The known genes or biological pathways involved in adaptation to the environment in the habitat of each group were determined. For instance, the YG group is distributed on the Yun-gui plateau (Southwest China), a low-latitude (∼23.3–26.6° N) and high-altitude (∼2000 m) region with acidic soil (pH 4.5∼5.5) (Supplemental Table S3). Genes related to metal ion (including potassium, iron, and zinc) binding and transport, cell membrane function, and response to toxins were overrepresented in this group (107 genes, adjusted *P* < 0.05) (Supplemental Fig. S3A), consistent with functions in overcoming cation deficiency and aluminum toxicity that are common in acidic soils (Seguel et al. 2013). For the YT group, we observed high enrichments of the LRR domain (24 genes), NB-ARC domain (eight genes), and other genes related to stress responses (32 genes; adjusted *P* < 0.05), in comparison to other groups, which was related to the strong selective pressures in high temperature and high humidity areas in middle and lower regions of the Yangtze river (annual average temperature 18°C, annual average air humidity >70%, annual precipitation >1400 mm) (Supplemental Table S3).

### Genome-wide environmental association studies of 51 environmental variables

**Figure 3.**
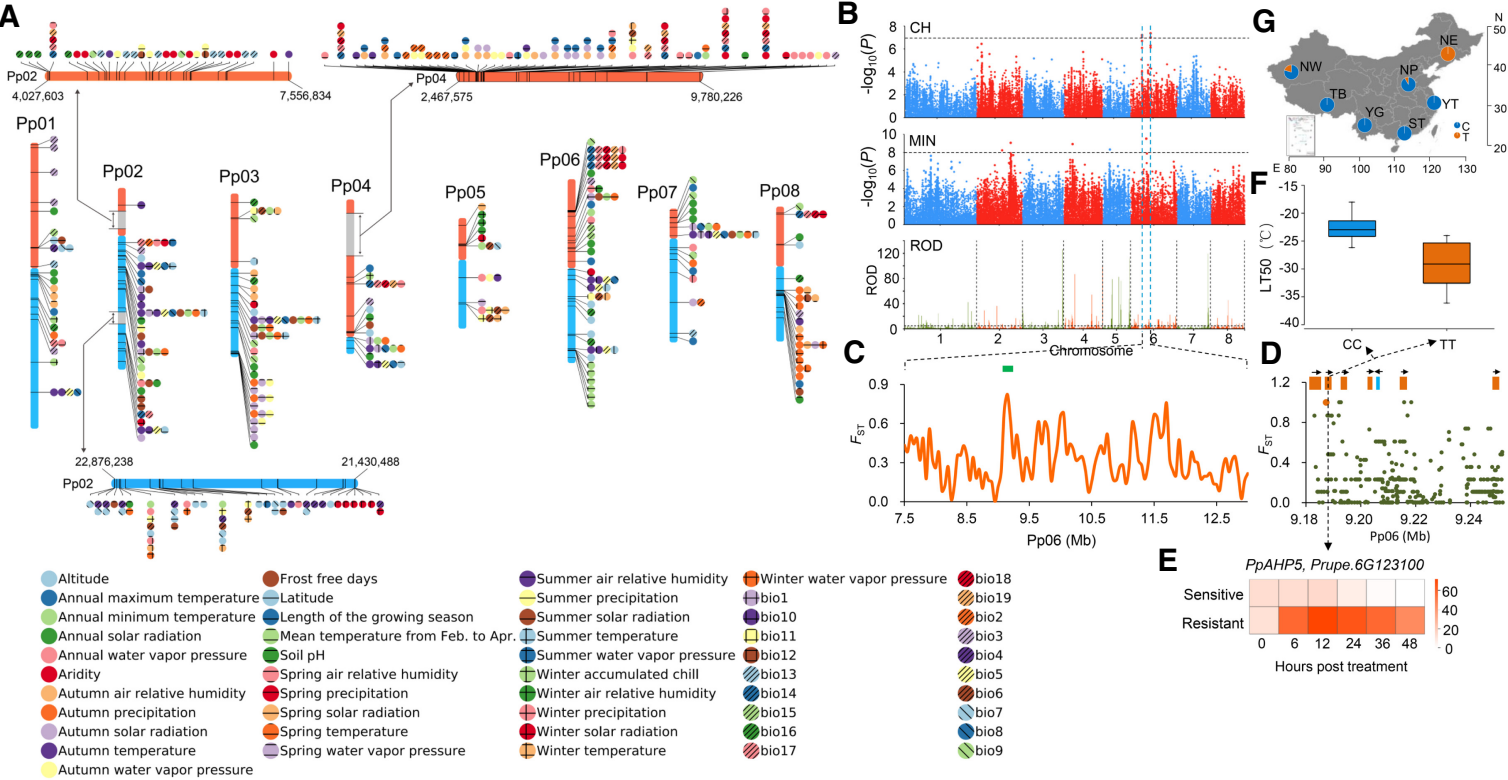
Genome-wide environmental association studies of 51 environmental variables and genomic loci associated with winter cold adaptation. (*A*) SNPs associated with environmental variables (EVs). Only the top 10 association signals for each EV are shown. All signals were included if the total number of signals was <10. Three EV association hotspots are highlighted using gray rectangles, and zoom-in figures for these hotspots are displayed. (*B*) The *PpAHP5* locus involved in adaptation to winter low temperature in peach. Manhattan plots for a GWAS study of cold hardiness (CH) and winter lowest temperature (MIN), and selection signals of the NE group are presented. The horizontal dashed lines represent the significance threshold for each test. The candidate genomic region is highlighted between two dashed blue vertical lines. (*C*) Distribution of *F*_ST_ values between NE and ST groups in the candidate region. The green bar indicates the *PpAHP5* locus. (*D*) Close-up view of the *F*_ST_ values in a region corresponding to the green bar in *C*. This region contains six *PpAHP* homologs (orange) and one other gene (light blue). The candidate SNP is highlighted using an orange dot. (*E*) Relative expression changes of *PpAHP5* after cold treatment (−28°C) in resistant and sensitive cultivars. (*F*) Associations between genotypes (CC or TT) of locus Pp06: 9,187,362 and cold hardiness (lethal temperature of 50%, LT50). (*G*) Allele (C or T) frequencies of association locus (Pp06: 9,187,362) in *PpAHP5* across the seven groups.

Although we obtained candidate genes underlying adaptation by identifying selective sweeps, many adaptive events in natural populations may occur by polygenic adaptation, which would be largely undetected by conventional methods for detecting selection (Pritchard and Di Rienzo 2010). However, local adaptation can generate correlations between environmental variables (EVs) and genomic loci which can be used to detect polygenic adaptation. We investigated a total of 51 EVs of the geographic origin of each accession that are important for plant adaptation (Supplemental Tables S6, S7), representing extremes and seasonality of temperature and precipitation, latitude, altitude, relative air humidity, water vapor pressure, growing season lengths, and radiations. Using a Mantel test, we found a significant correlation between environmental and genetic distances (Mantel statistic *r* = 0.38, *P* = 0.0001), with most associations being driven by altitude. To obtain loci associated with EVs, we performed GWEAS on 51 EVs. A total of 2755 association SNPs, involving 2408 genes, were identified (Fig. 3A; Supplemental Table S8). Overall, we found three EV association hotspots at the top and bottom of Chromosome 2 as well as at the top of Chromosome 4 (Fig. 3A). The top of Chromosome 2 has been reported to be enriched with genes encoding NBS-LRR proteins (Verde et al. 2013). We found that the hotspots at the bottom of Chromosome 2 and top of Chromosome 4 were highly enriched with genes associated with responses to a series of stresses and encoding LRR domain-containing proteins, respectively. Consistent with the high correlations among some climate variables (Supplemental Fig. S4), only 1670 association SNPs were unique, and ∼51.9% of the associations were shared across different types of EVs, suggesting that different EVs may shape the same genomic regions. A total of 75 genomic loci associated with more than five EVs were identified, and these loci were highly enriched with genes in stress-related pathways, such as plant–pathogen interaction, MAPK signaling pathway, response to stress, and defense response (adjusted *P* < 0.05) (Supplemental Table S9).

Next, we identified known biological processes that were overrepresented among associations for each EV and for overall EVs (Supplemental Table S10). Genes involved in ion transport were highly enriched in those associated with soil pH (adjusted *P* < 0.05), as soil pH affects absorption of metal ions in plants (Harter 1983). We found that functional categories related to response to a series of abiotic or biotic stimuli, programmed cell death (PCD), innate immune response, plant–pathogen interaction, and DNA repair were highly overrepresented (adjusted *P* < 0.05), suggesting that EVs have shaped genomic regions related to stress responses. A series of processes involved in secondary metabolism, including flavonoid metabolic process, jasmonic acid (JA) biosynthesis, and plant hormone signal transduction, were also significantly overrepresented (adjusted *P* < 0.05) (Supplemental Table S10). For instance, we found that genes related to JA biosynthesis were enriched in altitude associations (adjusted *P* < 0.05) (Supplemental Table S10). Previous study has shown that JA treatment contributes to enhanced cold resistance by promoting expression of the ICE-CBF/DREB1 transcriptional pathway, whereas a mutation in a key JA biosynthesis gene, *LOX1* (*Prupe.6G324400*, an altitude association gene in this study), leads to cold hypersensitive phenotypes (Hu et al. 2013).

Temperature and precipitation are two of the most important EVs that affect plant distribution and survival. We identified temperature-associated SNPs and found 10 association hotspots on Chromosomes 1, 2, 3, 4, 5, 6, and 7 for more than eight temperature-related EVs and altitude (Supplemental Fig. S5A,B). Tolerance to low temperature in winter is a major factor that restricts the spread of peach to extremely cold regions (north of 40°N). To characterize genetic loci underlying adaptation to extremely cold climates in peach, we performed a GWAS analysis of cold hardiness and identified four association peaks, on Chromosomes 2, 4, 6, and 7 (Fig. 3B). Of these, the peak on Chromosome 6 showed a strong selection signal, with sharp ROD in the NE group that experienced an extremely cold winter (lowest temperature <−30°C) (Fig. 3B). Moreover, this peak overlapped with the temperature association hotspot on Chromosome 6 and association peaks of annual lowest temperature (Fig. 3B; Supplemental Fig. S5B). The NE group (*n* = 19) inhabits areas north of 40°N that have extremely low winter temperatures (<−30°C), whereas the ST group (*n* = 14) grows in a contrasting climate, south of 25°N in areas with a warm winter (lowest temperature >10°C). We searched for genomic regions and SNPs with extremely high differentiation between ST and NE groups. One (Pp06: 9,187,362) of these SNPs (*F*_ST_ = 1) resided within the overlapping intervals between annual lowest temperature and cold hardiness associations (Fig. 3C). This SNP was located in the gene *PpAHP5* (*Prupe.6G123100*), belonging to a gene cluster encoding six histidine phosphotransfer proteins (AHP) (Fig. 3D), which have been reported to be involved in mediating cold signaling (Jeon and Kim 2013). Using cold treatment, we found this gene was up-regulated by cold, and the resistance cultivar harbored significantly higher expression levels of *PpAHP5* than the sensitive one (Fig. 3E). At this SNP locus, all representative accessions in the NE group showed a distinct genotype (TT) compared with the ST group (CC) (Fig. 3F), indicating that the TT genotype in *PpAHP5* is favored in high-altitude cold regions (Fig. 3G) and that *PpAHP5* is a candidate for conferring cold resistance in peach. We also detected six association hotspots for precipitation-related EVs, including annual and seasonal precipitation, length of growing season, aridity, and relative air humidity (Supplemental Fig. S5C,D). An extremely strong association hotspot on Pp02 (5.0∼7.2 Mb) was identified (Supplemental Fig. S5D), exhibiting enrichments of *R* genes (Verde et al. 2013), *RLKs* superfamily genes, NB-ARC domains, and other stress response-related genes, suggesting a genetic basis for precipitation adaptation.

To further elucidate the pattern of adaptation, we detected overlaps between selective sweeps and GWEAS signals, which resulted in a total of 639 shared genes (∼26.7% of GWEAS genes) (Supplemental Fig. S6). This revealed that, although selective sweeps are important, adaptations from standing variation or polygenic adaptation are also likely an important mode of adaptation in peach, which may be related to its short spread history after domestication (Li et al. 2019). These findings suggest that domesticated fruit species, such as peach, are generating and enhancing adaptation by standing selection on existing multiple sites. This situation is different from *A. thaliana*, which may have reached its adaptive limits owing to the constraints imposed by the limited generation of new mutations (Hancock et al. 2011). Collectively, these results indicate that both selective sweeps and GWEAS signals are central factors in the adaptive genetics of domesticated species.

### Adaptation to high drought regions

**Figure 4.**
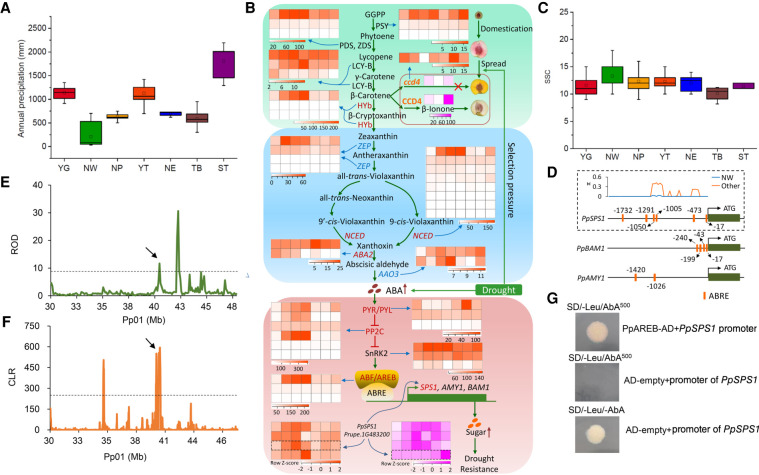
Genetic basis of drought resistance and high sugar content in the NW group. (*A*) Annual precipitation among geographic regions of the seven groups. (*B*) Relationship between the ABA pathway, drought stress, and evolution of flesh color. Heat maps in orange indicate gene expression levels (FPKM) under drought stress (0 h, 6 h, 12 h, 24 h, 3 d, 6 d, 12 d). Heat maps in pink indicate gene expression levels (FPKM) during peach fruit development (10, 50, and 90 d post-bloom date [dpb] for *PpCCD4*; 20, 40, 60, 80, 100, 120 dpb for *PpSPS1*). Genes under selection in the NW group are highlighted in red. Red arrows indicate the increase in levels of ABA and sugars. (*C*) Soluble solid content (SSC) among the seven groups. (*D*) ABRE *cis*-acting elements in the promoters of *PpSPS1*, *PpBAM1*, and *PpAMY1*. Orange boxes indicate ABRE elements in the promoter of each gene. The number around each ABRE represents the position from the ATG. The distribution of ABRE elements and nucleotide diversity (π) in the promoter of *PpSPS1* in the NW and other groups are shown in a dashed box. (*E*) Distribution of ROD around *PpSPS1* on Chromosome 1. Black arrow points to *PpSPS1*. (*F*) Distribution of CLR values around *PpSPS1* on Chromosome 1. Black arrow points to *PpSPS1*. (*G*) Verification of the interaction between *PpAREB* (*Prupe.1G434500*) and the promoter of *PpSPS1* (*Prupe.1G483200*) using a yeast one-hybrid assay.

The NW group is from northwestern China, which has an extreme climate, characterized by severe aridity (<150 mm annual rainfall) (Fig. 4A) and extreme high or low temperatures in the summer (>40°C) or winter (<−30°C) (Supplemental Table S3). Peach accessions from this region are frequently challenged by high drought stress. We found that genes overrepresented in this group included those involved in abscisic acid (ABA) biosynthesis and signal transduction (adjusted *P* < 0.05) (Supplemental Fig. S3B), which are well known to regulate drought stress responses. Transcriptome analyses of peach accessions grown under drought stress conditions revealed that genes involved in the ABA pathway were highly enriched among differentially expressed genes (DEGs), including *NCED*, *PYR*, *ABA2*, *PP2C*, and *ABRE* genes that showed selective signals in the NW group (Fig. 4B), further suggesting a key role of the ABA pathway in peach drought responses.

Sugars function as important signaling molecules in response to a range of abiotic and biotic stresses in plants (Lastdrager et al. 2014). We found that peach fruits produced by accessions from the NW group, especially those from Xinjiang province (Wang et al. 2012), consistently had higher soluble sugar content (SSC) than those from other groups (Fig. 4C). Associated long-term natural selection pressures contributing to greater accumulation of soluble sugars likely include aridity, high diurnal temperature variation, and long sunshine duration. Moreover, the starch and sucrose metabolism pathways were overrepresented in both DEGs under drought stress treatment (35 genes) and genes under selection in the NW group (12 genes; adjusted *P* < 0.05) (Supplemental Fig. S3B), congruent with roles of sugars in drought stress. Furthermore, all the 12 genes in selective sweeps were differentially expressed following the drought stress treatment. We hypothesize that higher soluble sugar content in accessions from northwestern China represents an adaptive trait driven by the local drought environment.

Previous studies of apple have demonstrated that drought stress and ABA contributed to soluble sugar accumulation through the activation of sugar transporter and amylase genes by the ABA-responsive transcription factor, *AREB2* (Ma et al. 2017). Similarly, both drought stress and exogenous ABA induce an increase in soluble sugar accumulation in peach fruit (Kobashi et al. 2000, 2001). Here, we found that two putative gene targets of AREB2 (Fig. 4B,D), *PpAMY1* (*Prupe.1G142400*) and *PpBAM1* (*Prupe.1G053800*), were up-regulated by drought treatment; however, neither exhibited a significant selection signal. To identify additional target genes in drought-mediated sugar accumulation, we searched for genes harboring the putative binding motifs of AREB2 among genes under selection in the NW group. This revealed a sucrose phosphate synthase gene (*PpSPS1*, *Prupe.1G483200*), with six ABA-responsive elements (ABREs) in the promoter region (Fig. 4D), showing a strong selection signal, with high ROD and composite likelihood ratio (CLR) values (Fig. 4E,F). *PpSPS1*, which is involved in the biosynthesis of sucrose, the predominant soluble sugar in mature peach fruit and the key factor conferring sweetness, was up-regulated by drought treatment (Fig. 4B), suggesting its roles in drought stress response. The expression of *PpSPS1* was increased by ∼500-fold during fruit maturity (Fig. 4B), implying its roles in fruit ripening and sugar accumulation. Using a yeast one-hybrid experiment (Supplemental Methods), we verified the interactions between AREB/ABF and the promoter of *PpSPS1* (Fig. 4G), providing new insight into ABA-mediated enhanced sugar accumulation under drought stress. The selection on sugar-related genes may mediate adaptation to drought stress in the NW group, accompanied by the increases in fruit sugar content. In addition, we found that the top of Chromosome 5 and the middle of Chromosome 4, which have been reported to harbor major SSC- and sugar content-associated quantitative trait loci (QTLs) and SSC candidate gene *PpNCED3* (Martínez-García et al. 2013; Li et al. 2019), also showed strong selection signals in the NW group. Selections on these genes may underlie the genetic basis of high sugar levels in peaches grown in areas with high drought stress.

We found that the flesh color of peach showed strong geographic patterns, with ∼80% of yellow-fleshed peach landraces originating from northwestern China (NW group). Yellow flesh of peach mainly depends on the content of carotenoids at maturity, including β-cryptoxanthin and β-carotene, and carotenoids are believed to be the major precursors for ABA biosynthesis (Fig. 4B). A previous study identified three loss-of-function variants involved in a carotenoid cleavage dioxygenase gene (*PpCCD4*, *Prupe.1G255500*), leading to the abnormal carotenoid degradation and yellow flesh (Falchi et al. 2013). The disturbed function of *PpCCD4* in yellow-fleshed peach resulted in the retention of carotenoids, which can provide more precursors for ABA biosynthesis (Fig. 4B) and may contribute to higher ABA levels and subsequent enhanced drought tolerance. Moreover, we found that *PpCCD4* was down-regulated by drought treatments (Fig. 4B). Furthermore, the carotenoid biosynthetic pathway was highly overrepresented with genes under selection in the NW group (adjusted *P* < 0.05). Therefore, we hypothesize that yellow peach flesh has undergone long-term adaptive selection, driven by drought stress, and that modern yellow-fleshed peach cultivars may originate from northwestern China.

Collectively, we constructed a joint pathway for drought adaptation evolution in peach (Fig. 4B), driven by the complicated interactions between carotenoids, ABA, and sugar, of which ABA may be the central controller and play the key role.

### Adaptation to high altitudes

**Figure 5.**
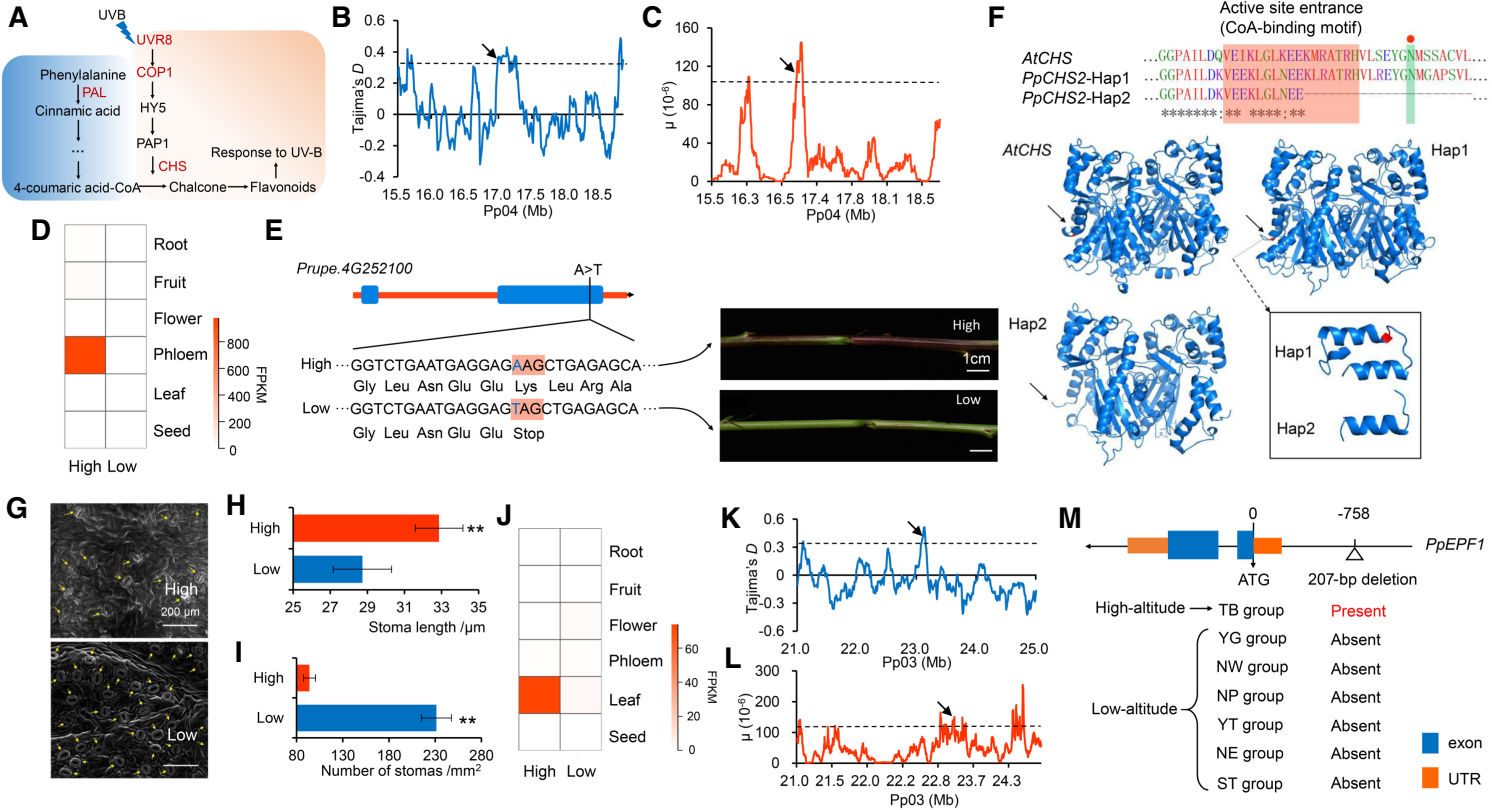
Genomic regions and candidate genes related to high-altitude adaptation of peach. (*A*) Pathway related to plant response to UVB. Genes under selection are highlighted in red. (*B*,*C*) Distribution of Tajima's *D* (*B*) and μ statistic (*C*) in the region around *PpCHS2* (*Prupe.4G252100*) on Chromosome 4 (15.5–19.0 Mb). The dashed horizontal lines indicate a threshold of the top 5% for Tajima's *D* (≥0.36) and μ test (≥1.07). Arrows point to *PpCHS2.* (*D*) Heat map of expression profiles of *PpCHS2* in different tissues in low- and high-altitude accessions. (*E*) A candidate stop-gained SNP in *PpCHS2* that is associated with high-altitude adaption and new shoot colors in accessions from low and high altitudes. (*F*) Effects of stop-gained SNP on protein structure of CHS. 3D structure of CHS protein was obtained from Swiss-Prot. The red shadow represents the CoA-binding motif. The green shadow represents one of the conserved enzyme active site. (*G*) Scanning electron microscopy (SEM) of stomata from leaves of high- and low-altitude accessions. The magnification is 800×. (*H*,*I*) Stomatal length (*H*) and stomatal density (*I*) in high- and low-altitude accessions. (**) *P* < 0.01. (*J*) Heat map of expression profiles of *PpEPF1* in different tissues in accessions from low and high altitudes. (*K*,*L*) Distribution of Tajima's *D* (*K*) and μ values (*L*) in a region around *PpEPF1* (*Prupe.3G235800*) on Chromosome 3 (21.0–25.0 Mb). The dashed horizontal lines indicate a threshold of the top 5% for Tajima's *D* (≥0.36) and μ test (≥1.07). Arrows point to *PpEPF1*. (*M*) Structure of *PpEPF1* and the position of the 207-bp deletion. The presence and absence of the 207-bp deletion in the seven groups are given.

Members of the TB group (*n* = 45) are from “the roof of the world”, the Tibet plateau, which is the highest plateau on the earth, with an average elevation of 4500 m. This area is inhospitable to many organisms because of its strong ultraviolet radiation, hypoxia, and severe cold (Supplemental Table S3). At high altitudes, genome integrity is continuously challenged by intensive solar ultraviolet radiation (UVB, 280–315 nm)-induced DNA damage. Peach accessions in the TB group tolerate these conditions using several adaptation-related phenotypes, such as a dark branch color, epigeal germination, and red-colored new shoots (Supplemental Fig. S7). We identified 339 genomic regions, harboring 743 genes, showing signals of natural selection in the TB group (Supplemental Table S4). Of these, we found a significant enrichment of genes involved in response to the UVB category (adjusted *P* = 0.000397) (Supplemental Fig. S3F), which is consistent with adaptation to the high-altitude origin of the TB group. Flavonoids are a group of plant secondary metabolites which play important roles in UVB protection (Li et al. 1993), and we found two genes in the flavonoid biosynthetic pathway in the “response to UVB” category (Fig. 5A): chalcone synthase 2 (*PpCHS2*, *Prupe.4G252100*) and phenylalanine ammonia-lyase (*PpPAL*, *Prupe.6G235400*), both of which showed strong selection signals in the TB group, with high μ and Tajima's *D* values (Fig. 5B,C). Chalcone synthase catalyzes the first committed step in flavonoid biosynthesis, and previous studies showed that functional perturbation of an *A. thaliana* homolog, *CHS*, resulted in UV-hypersensitive phenotypes, while *CHS* was up-regulated in a UVB-tolerant mutant (Bieza and Lois 2001). We found that *PpCHS2* was highly and specifically expressed in the phloem of new shoots in the TB group (Fig. 5D), consistent with the red new shoot phenotype. By scanning genomic variants in or around *PpCHS2*, we found that a SNP (Pp04: 16,896,126, A > T) causing the introduction of a premature termination codon (Fig. 5E) showed a high frequency in low-altitude accessions (76.3%) but an extreme low frequency in the TB group (2.0%). This SNP was located at the CoA-binding motif (Fig. 5F), the key active region for protein function, leading to an incomplete binding motif that may result in the loss of function. Moreover, the premature termination resulted in the loss of one conserved catalytic residue which is also crucial for the catalytic activity (Ferrer et al. 1999). Therefore, this SNP was designated as a candidate causative variant for the red new shoot phenotype involved in flavonoid-mediated UVB adaptation. Collectively, our results suggest that selection on the *CHS2* gene and the regulation of anthocyanins may be one of the important mechanisms to confer tolerance to the damage from UV irradiation for peach at high altitudes.

We observed that, compared to low-altitude accessions, those from high altitudes had a lower density of stomata and larger stomata size (Fig. 5G–I; Supplemental Methods). This may represent an adaptive evolution to hypoxia at high altitudes. We found that the biological category stomatal complex patterning was significantly enriched in the gene set under selection (adjusted *P* = 0.00081). One of the genes in this category, *Prupe.3G235800*, was highly and specifically expressed in leaves, showing an altitudinal pattern with higher expression levels in the TB group than in the low-altitude group (Fig. 5J). *Prupe.3G235800*, which encodes the epidermal patterning factor 1 (PpEPF1) involved in stomatal development (Hara et al. 2009), showed strong selection signals, based on the high Tajima's *D* and μ values (Fig. 5K,L). By scanning the variants in *PpEPF1*, we found that SNPs with functional significance were absent. Through further scanning variants upstream of or downstream from *PpEPF1*, we identified a TB group-specific 207-bp deletion in the promoter region of *PpEPF1* (Fig. 5M), suggesting that the adaptive evolution controlled by *PpEPF1* may be mediated by regulation of its expression. Furthermore, overexpression of *PpEPF1* in *A. thaliana* resulted in a decrease in stomatal density (Supplemental Fig. S8; Supplemental Methods). Moreover, the *epf1* mutation in *A. thaliana* resulted in increased stomatal density (Hara et al. 2009). These results suggest that selection on *PpEPF1* may be closely related to adaptation to hypoxia in high altitudes through regulating stomatal density.

### A major *SVP* locus involved in adaptive evolution of bloom date

**Figure 6.**
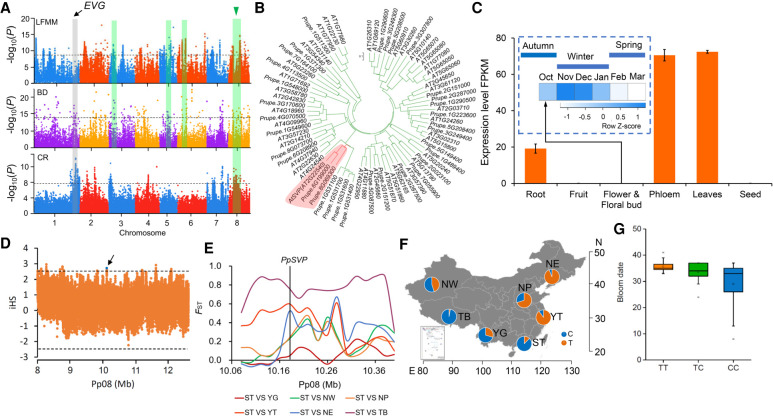
A major *PpSVP* locus involved in local adaptation of bloom date in peach. (*A*) Manhattan plots of SNPs associated with EVs (LFMM), bloom date (BD), and chilling requirement (CR). Dashed horizontal lines represent the significance thresholds for the tests. The overlapped regions between GWAS for BD and LFMM are highlighted using green shaded rectangles. The major QTL for CR and BD overlapping with local selection signals on Chromosome 8 surrounding *PpSVP* is indicated by a blue triangle. The *EVG* locus is highlighted using a gray shaded rectangle. (*B*) Neighbor-joining tree of *PpSVP* and MIKC-type MADS family genes. The clade containing *PpSVP* is highlighted in red. (*C*) Temporal and spatial expression patterns of *PpSVP*. Error bars represent standard deviation of three biological replicates. (*D*) Patterns of normalized iHS scores across the ∼4.5-Mb genomic region around *PpSVP*. The dashed horizontal lines represent the threshold of positive selection signal (|iHS| > 2.5). The blue dot indicates the SNP (Pp08: 10,173,576) that showed high iHS score in *PpSVP*. (*E*) *F*_ST_ around *PpSVP* among different groups. The associated SNP in *PpSVP* is indicated using vertical black line. (*F*) Allelic frequencies of the associated SNP (Pp08: 10,173,576) in *PpSVP* across the seven groups. C and T represent the alleles of this SNP. (*G*) Relationship between genotypes of associated SNP (Pp08: 10,173,576) and bloom date. TT, TC, and CC represent the three genotypes of this SNP.

Bloom date (BD) is crucial for local adaptation in peach and is controlled by multiple genes (Fan et al. 2010). To explore the genetic basis of adaptation of BD, we performed GWAS of BD using 174 accessions that were phenotyped. This revealed 399 associated SNPs and 12 association peaks (Fig. 6A), of which six overlapped with previously reported QTLs (Fan et al. 2010). Next, we identified candidates involved in local adaptation by detecting SNPs showing associations with EVs (PC1 for 51 EVs, explained ∼60% variation) using a latent factor mixed-effect model (LFMM) (Frichot et al. 2013; Wang et al. 2018), resulting in a final set of 23 association peaks (Fig. 6A). By overlapping BD GWAS and LFMM analyses, we found four regions on Chromosomes 3, 5, 6, and 8 that may underlie the local adaptation of BD during the spread of peach to different climates (Fig. 6A).

Chilling requirement (CR) is another important adaptive trait and is significantly correlated with BD. We reperformed the GWAS for the CR based on our previous study (Li et al. 2019) using 174 landrace accessions and identified six association peaks, of which three (Chromosomes 1, 7, and 8) were shared with BD GWAS loci (Fig. 6A), including the major QTL for CR harboring the *EVG* locus conferring dormancy mutation in peach (Li et al. 2009). After overlapping GWAS signals of CR and BD with the LFMM analysis, we found a strong overlap spanning ∼4 Mb on Chromosome 8, which may be important for local adaptation of BD in peach (Fig. 6A). However, the major QTL for CR and BD on Chromosome 1 showed no local adaptation signal in the LFMM analysis (Fig. 6A), suggesting that climates may drive the evolution of BD and CR by shaping QTLs with small effects.

The 4-Mb region encompasses 275 genes, including a putative ortholog of *A. thaliana SHORT VEGETATIVE PHASE* (*PpSVP*, *Prupe.8G069300*). *SVP* is involved in controlling flowering time and has previously been implicated in regulating dormancy in *Prunus* (Li et al. 2009; Sasaki et al. 2011; Zhang et al. 2012a). Phylogenetic analysis confirmed that *PpSVP* belongs to a MADS-box family and is closely related to the *AGL22* subfamily (Fig. 6B). *PpSVP* showed strong tissue-specific expression, with high expression only in vegetative organs. Moreover, expression of *PpSVP* was up-regulated during dormancy induction and down-regulated by winter chill (0°C–7.2°C) and by forcing temperature (heat) in floral buds in spring (Fig. 6C), suggesting its potential roles in regulating BD and CR. Moreover, through calculating the standardized integrated haplotype score (iHS) for SNPs located in this overlap region, we also found a strong positive selection signal around the *PpSVP* locus (Fig. 6D). Additionally, an exceptionally high *F*_ST_ value was identified in this region, especially between the ST and NE groups and between the ST and YT groups (Fig. 6E) that harbor a distinct bloom date. The *PpSVP* locus thus represents a strong candidate gene for local adaptation of BD and CR. We propose that spatially varying selection has driven latitudinal differentiation at this locus. Overall, all these results provide compelling evidence of local selection on the *PpSVP* locus during adaptive evolution to different climates after domestication.

To identify the causal variants underlying adaptation of BD, we screened for SNPs with high *F*_ST_ between the NE (late bloom) and ST (early bloom) groups at the *PpSVP* locus. No SNP with high differentiation was identified that caused an amino acid change. However, a SNP located at 5′-untranslated regions with a high *F*_ST_ value (*F*_ST_ = 0.9) was identified, suggesting that the BD and CR may adapt to different climates through shaping the expression of the controlled gene. Allele frequencies of this SNP showed a strong geographical pattern and the early bloom allele (CC) was mainly present in groups from low altitudes (ST and YG groups) and the wild group (TB group) (Fig. 6F,G), consistent with their phenotypes. This also provides insights into two distinct evolutionary routes of BD and CR in low- and high chill regions. Moreover, a previous study reported that overexpression of the low-altitude favored genotype of *PpSVP* in *A. thaliana* resulted in plants with strong vegetative growth and delayed flowering time (Li et al. 2019).

### Genomic locus associated with response to climate change

**Figure 7.**
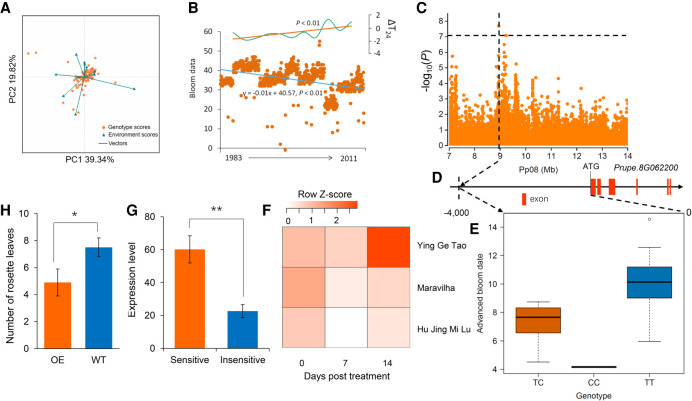
Genotype–environment interaction analysis and genome-wide association study of advance in bloom date. (*A*) Genotype–environment interaction analysis of bloom date from 1983 to 2011 using the AMMI analysis. (*B*) Scatterplots of relative bloom date of 89 peach accessions from 1983 to 2011 and temperature change in the spring. The blue and orange lines represent the trend of bloom date changes and temperature changes in the spring, respectively, based on the linear regression analyses. ΔT24 indicates anomalies in the mean temperature from February to April compared to those from 1983 to 2011. (*C*) Regional Manhattan plot of GWAS for ABD on Chromosome 8 of the 7.0- to 14.0-Mb region. The horizontal black dashed line indicates significance threshold (*P* < 7.28 × 10^−8^ or −log_10_[*P*] > 7.08) using a Bonferroni test (0.05). (*D*) The most significant SNP associated with ABD and its location relative to gene *PpLNK1* (*Prupe.8G062200*). (*E*) Association between genotypes of the most significant SNP and ABD. (*F*) Changes in *PpLNK1* expression in three cultivars in a climate-warming simulation experiment. (*G*) Comparison of *PpLNK1* expression between accessions sensitive and insensitive to global warming at blooming. (**) *P* < 0.01. (*H*) Comparison of BD between wild type (WT) and *PpLNK1* overexpression (OE) *A. thaliana* lines. (*) *P* < 0.05.

Adaptation to accelerating rates of climate change is increasingly important for species survival. The advance in bloom date (ABD), as a consequence of global warming over recent decades, has been observed in many temperate species, including peach (Menzel et al. 2006; Li et al. 2016). However, the genetic mechanisms underlying ABD have not been characterized. We performed a long-term observation of BD with 89 peach accessions spanning three decades, from the 1980s to the 2010s (Supplemental Fig. S9A). We observed a significant ABD (*P* < 0.001), based on an additive main effects and multiplicative interaction (AMMI) analysis of multiple year BD data (Annicchiarico, 1997), and the main driver was found to be a temperature rise in the spring (explained 61.3% of the variation, *P* < 0.001) (Fig. 7A). Using a linear regression analysis, we quantified ABD and found that BD has advanced by ∼10 d on average over the last 30 yr (Fig. 7B).

Next, we performed GWAS for ABD to identify genetic loci associated with responses to global warming (Supplemental Fig. S9B). This revealed a strong association peak on Chromosome 8 (*P* < 7.28 × 10^−8^) (Fig. 7C) in an area harboring 14 candidate genes. This association was also located at an overlap among GWAS signals of CR, BD, and LFMM analysis. The most significant SNP was located in a region upstream of *Prupe.8G062200*, with the genotype of TT showing as sensitive to global warming and CC as insensitive (Fig. 7D,E)*. Prupe.8G062200* encodes a putative NIGHT LIGHT-INDUCIBLE AND CLOCK-REGULATED 1 (LNK1) protein and showed high expression levels at blooming. A homolog of this gene in *A. thaliana* is involved in regulation of the circadian clock, which regulates *COL1* genes at warm temperatures and is thus a potential regulator of flowering time (Mikkelsen and Thomashow 2009; Rugnone et al. 2013). A simulation experiment showed that *PpLNK1* was up-regulated by rising temperatures during heat accumulation, suggesting that *PpLNK1* may be up-regulated by temperature rise in spring (Fig. 7F). In addition, expression of *PpLNK1* in peach accessions that are sensitive to global warming was significantly higher than in those that are insensitive (Fig. 7G). Moreover, overexpression of *PpLNK1* in *A. thaliana* led to early flowering (Fig. 7H; Supplemental Methods). Furthermore, several *cis*-elements associated with temperature and light responsiveness were identified (Supplemental Table S11). Therefore, we conclude that *PpLNK1* may play important roles in regulating the annual circadian clock of flowering time as influenced by rising temperature in peach. *PpLNK1* is thus a plausible candidate gene for responses to global warming, which can improve our understanding of the genetic architecture of plant adaptation to global climate change, but further work will be necessary to provide more direct evidence of its roles.

Long-term observation of BD enabled multiyear GWAS. We identified a total of 713 SNPs associated with BD (*P* < 7.28 × 10^−8^), including 483 temporary associations that were identified only in 1 yr, 214 associations in at least 2 yr, and 16 stable associations in more than 5 yr, of which several overlapped with previous reported QTLs (Supplemental Table S12; Fan et al. 2010). Among stable associations, a strong association peak within a small intergenic region (Pp06: 15,327,714–15,354,080) on Chromosome 6 was identified in 8 yr of GWAS, which can be further developed for marker-assisted selection.

## Discussion

Plant genomes have been shaped by natural selection during local adaptation to diverse environmental conditions. Adaptation is closely related to species survival and response to changing climates. Natural populations of peach provide an excellent model to investigate the genetic bases and modes of adaptation to different climates, thanks to its relatively small genome size and extensive climatic gradients across its native range. Here, we generated a large variation map for a climate-extensive panel of 263 peach landraces and wild relatives. The variation pattern revealed the significant geographic structure and the adaptive genomic footprints shaped by environments.

Understanding the genetic bases of adaptation to extremely harsh environments is essential for survival in future global climate change. A series of studies have predicted the extreme climate conditions in the future (Reich et al. 2018; Bastin et al. 2019; Xu et al. 2020), such as high temperature, aridity, sea level rise, flood, soil salinization, severe cold, etc. In this study, we elucidated the genome-wide genetic patterns of adaptation to high altitude, high drought, and severe cold, using peach wild relative or landrace accessions. Several key candidate genes were identified, including *PpCHS2*, *PpEPF1*, *PpSPS1*, and *PpAHP5*. Many studies have revealed the roles of sugars in drought tolerance, such as in apple (Ma et al. 2017), grape (Ferrandino and Lovisolo 2014), and rice (Akihiro et al. 2005), and sugar biosynthesis- and transport-related genes participate in sugar-mediated drought tolerance. We found that a peach sucrose biosynthesis-related gene, *PpSPS1*, showed a strong selection signal in a high drought region and was involved in the sugar- and ABA-mediated drought tolerance pathway. *Prunus mira* (TB group) is the largest population of fruit trees on the highest plateau in the world, the Qinghai-Tibet plateau. Although they are continuously exposed to strong UV radiation, hypoxia, and severe cold, many trees have lived for hundreds of years, some more than 1000 yr (Supplemental Fig. S7D). In addition to the key roles of anthocyanins, we also found that genes involved in DNA repair and response to DNA damage were highly overrepresented in the TB group (adjusted *P* < 0.05) (Supplemental Fig. S3F), which may underlie the long lifespan of *P. mira*. Collectively, although harsh environments have strongly challenged the survival of peach, the adaptive evolution and adaptive variation generated under stress pressure may provide excellent materials for breeding.

Selective sweeps are powerful in identifying the adaptive alleles at high frequency. However, many adaptive events in natural populations may occur by polygenic adaptation, which would largely stay undetected by detecting selective sweeps (Pritchard and Di Rienzo 2010). GWEAS is an important complement to selective sweeps in the analytical interpretation of species adaptation. In *Arabidopsis* and sorghum, environment associations have been successfully used to predict fitness using common environment experiments (Hancock et al. 2011; Lasky et al. 2015). In this study, genetic bases of peach adaptation were revealed by the combination of selective sweeps and environmental associations. We found that both environment associations and selective sweeps played critical roles in peach adaptation. Therefore, for peach, the models for predicting the relative fitness and genome–environment interactions trained based on the integrated adaptive signals are more accurate and comprehensive.

Ongoing anthropogenic climate change is having marked impacts on the Earth, including the loss of biodiversity, decline of food production, and contraction of distribution range (Scheffers et al. 2016). Integrating population genomics and environmental data is useful to assess the ability to adapt to climate change. Bay et al. (2018) combined genomic data and global climate predictions to develop a powerful tool for estimating the potential to adapt to climate change and predict future population declines of yellow warblers by genomic vulnerability score analyses. In this study, the impacts of climate change on one of the most important adaptive traits of peach, bloom date, were artificially assessed using a 30-yrs investigation, from the 1980s to the 2010s. Similarly, these data, combined with genomic data, gave the opportunity to identify a candidate gene (*PpLNK1*) in peach associated with response to climate change. In addition, we could also understand the genotypes that are insensitive or sensitive to climate change, which is useful for assessing the adaptive ability to climate change of peach. However, a prediction model based on only one adaptive trait is limited, especially for perennial woody crops. In the future, phenomics-based long-term investigation and multi-omics data may be more powerful to train the exact model to predict the response to climate change of agricultural crops.

In summary, this study provides new insights into peach adaptation to its habitat and how climate has shaped the genome of a perennial tree through natural selection. These results also provide a new resource for studies of peach evolutionary biology and breeding, especially with regard to enhancing stress resistance.

## Methods

### Plant materials and sequencing

A total of 263 peach accessions were used in this study, of which 218 were from the National Peach Germplasm Repository of China (NPGRC) and 45 *P. mira* accessions were sampled from the Tibet plateau. These accessions, collected from almost all the distribution regions of peach landraces and wild relatives in China, belonged to seven major ecotypes (Supplemental Table S1; Wang and Zhuang 2001), of which 260 have been reported in our previous study (Li et al. 2019) and three were newly sequenced. Total genomic DNA of the three newly sequenced accessions was extracted from young leaves using the cetyltrimethylammonium bromide (CTAB) method (Murray and Thompson 1980) and sequenced on the Illumina HiSeq 2500 platform (Illumina) with 125-bp paired-end reads (Supplemental Table S1; Supplemental Methods).

### Variation calling and annotation

Paired-end reads from each accession were mapped to the peach “Lovell” genome (release v2.0) using BWA (version: 0.7.12) (Li and Durbin 2009). After removing PCR duplicates and low-quality alignments, SNP and indel callings were performed using GATK HaplotypeCaller (McKenna et al. 2010) following the method described in a previous study (Li et al. 2019). SNP annotation was performed based on genomic locations and predicted coding effects, according to the peach genome annotation (release annotation v2.1), using SnpEff (version: 4.1g) (Cingolani et al. 2012). SV calling was performed using the SpeedSeq (Chiang et al. 2015), DELLY (Rausch et al. 2012), and Manta (Chen et al. 2016) programs following the method described in a previous study (Li et al. 2019). The details are available in Supplemental Methods.

### Population genetics analysis

A subset of 3,429,878 SNPs with MAF > 0.01 were used to construct a neighbor-joining tree using PHYLIP (version: 3.696) (Felsenstein 1989) with 1000 bootstrap replicates. The principal component analysis and population structure (K = 2-8, bootstrap 200) were investigated using SmartPCA (version: 6.0.1) (Price et al. 2006) and ADMIXTURE (version: 1.1) (Alexander et al. 2009) based on the same SNPs data set, respectively. The details are available in Supplemental Methods.

### Identification of select sweeps

To detect signals of selective sweeps of six landrace groups (YG, NW, NP NE, YT, and ST), we selected three distinct genome-wide selection metrics for each group, including the reduction of nucleotide diversity (π), Tajima's *D*, and genetic differentiation (*F*_ST_). To determine whether the poor assembly regions of the genome could cause the bias of ROD and *F*_ST_, we used the repeat regions (37.1% of genome) as the example of potential poor assembly regions and found that there was no significant difference in nucleotide diversity (π) (0.0018 in repeat regions vs. 0.0019 in nonrepeat regions), suggesting that the impacts of assembly quality on the identification of selective sweeps was very minor using the current version of the peach genome. Finally, we calculated these three selection metrics based on all SNPs (4,611,842) using VCFtools (version: 0.1.13) (Danecek et al. 2011), with a 10-kb window and a step size of 1 kb. ROD and *F*_ST_ were calculated between a specific group and the mix of the other five groups. We defined the empirical top 5% of windows or regions as candidate selective outliers for each selection scan metric. The adjacent selective outliers were merged. For each population, selection outliers detected in at least two of the selection scan metrics were designated as the candidate selection regions. The TB group consisted of wild relatives (*P. mira*), and three other methods were used to detect selective sweeps: Tajima's *D*, *μ* statistic (Alachiotis and Pavlidis 2018), and composite likelihood ratio, which were calculated using VCFtools, RAiSD (version: 1.7) (Alachiotis and Pavlidis 2018), and SweeD (version: 3.2.1) (Pavlidis et al. 2013), respectively, with a 10-kb sliding window. Similarly, the top 5% of windows or regions identified in at least two metrics were designated as candidate selective sweeps. To further verify the selection signal, we calculated the iHS scores for specific genomic regions using iHS module in the selscan program with default parameters (version: v1.2.0a) (Szpiech and Hernandez 2014). The final selective sweeps of seven groups were visualized using Circos (version: 0.69) (Krzywinski et al. 2009).

### Collection of climate variables

A total of 51 environmental variables were selected as being essential for peach growth and survival (Supplemental Table S6). These environmental variables data for each accession were collected from WorldClim (http://www.worldclim.org; version: 1.4), CMDC (http://data.cma.cn/en/?r=site/index), and FAO GeoNetwork (http://www.fao.org/geonetwork/srv/en/main.home) (Supplemental Table S6; Supplemental Methods).

### Genome-wide environmental association study

GWEAS was performed for 51 EVs using 211 landrace accessions based on 4,596,331 SNPs using the mixed linear model (MLM) with Efficient Mixed-Model Association eXpedited (EMMAX) software (Kang et al. 2010). The kinship matrix and PCA were used as the random effect and fixed effect covariates, respectively. The genome-wide significance threshold was set as 0.05/total number of SNPs (log_10_[*P*] = −7.13) using the Bonferroni test. Due to the high degree of correlations among these EVs (Supplemental Fig. S4), we performed a PCA on 51 EVs using the “prcomp” function in R (version: 3.3.4) to identify PCs that best summarized the range of environmental variation (R Core Team 2018). The PCA result showed that the first environmental PC (PC1) explained >60% of the total variance, which can represent our overall environmental variable. The LFMM analyses was performed with PC1 of EVs and genome-wide SNPs using R package lfmm (version: 1.3) with the following parameters: -p 8 -K 3 -I 10000 (Frichot et al. 2013).

### Functional enrichment and pathway analysis

Gene Ontology (GO) enrichment analysis was performed using agriGO (version: 2.0) based on Fisher's exact test with Bonferroni adjustment (Tian et al. 2017). Kyoto Encyclopedia of Genes and Genomes (KEGG) pathway enrichment analysis was performed using KOBAS (version 3.0) based on Fisher's exact test with QVALUE correction (Xie et al. 2011).

### Phenotyping and genome-wide association study (GWAS)

The first bloom date of peach was measured at NPGRC (Zhengzhou, Henan Province, China; N34.71°, E113.70°, A.S.L. 74 m) from February 25 to April 25 in the years of 1983 to 2011. A total of 89 accessions, with each represented by two replicates, were used to investigate BD (Supplemental Fig. S9A). The BD was defined as the day when ∼5% of the flowers have completely opened. The interactions between climate change and BD were determined by the AMMI analysis using the BD spanning ∼30 yr using Genstat software (version: 18; https://www.vsni.co.uk/software/genstat). The advanced days of bloom date from 1983 to 2011 were defined as the advance in bloom date. The ABD for each accession was estimated using a linear regression analysis, based on the BD from 1983 to 2011.

To identify genetic loci associated with the ABD, GWAS was performed using the EMMAX program (Kang et al. 2010) with a set of 873,895 SNPs with MAF > 0.05 and a data missing rate < 0.2. The kinship matrix and PCA were used as the random effect and fixed effect covariates, respectively. The genome-wide significance threshold was set as 0.05/total number of SNPs (−log_10_[*P*] = 7.08) using the Bonferroni correction. GWAS was also performed for yearly BD data from 1983 to 2011 with the same SNP data set, using the same method described above.

For CR, phenotyping analyses were performed in 2011 and 2012 as in Fan et al. (2010) and Li et al. (2019) on 174 landrace accessions. A 0°C –7.2°C model was chosen to evaluate CR, and GWAS for CR was performed using the same method with ABD (Supplemental Methods).

Cold hardiness was evaluated using a conductance-based semilethal temperature method described in Zhang et al. (2012b) in December–January of 2013–2014 on 143 accessions. A total of six cold treatments were used: −10°C, −15°C, −20°C, −25°C, −30°C, and −35°C. The semilethal temperature (LT50) was calculated using a logistic function based on the relative conductance. GWAS for cold hardiness was performed using the same method described above. The details are available in Supplemental Methods.

### RNA-seq analysis

For drought stress treatment, four-year-old potted peach seedlings from peach cultivar “Dong Xue Mi Tao” were used. Fruit flesh were taken at six drought stress treatment time points, including 6 h, 12 h, 24 h, 3 d, 6 d, and 12 d. For expression profiles in different tissues, roots, leaves, fruit, flowers, phloem, and seeds were sampled from “Aba Guang He Tao” (high-altitude) and “B-4” (low-altitude). For the expression of *PpCCD4,* fruit fleshes were sampled from “Zao Huang Pan Tao” (yellow-fleshed) and “Zhong Tao Hong Yu” (white-fleshed) at 10, 50, and 90 d post-bloom date (dpb). For the expression of *PpSPS1*, fruit fleshes were sampled from “Chinese Cling” at 20, 40, 60, 80, 100, and 120 dpb. Three biological replicates were collected for each sample. RNA-seq libraries were sequenced using the Illumina HiSeq 2000 platform (Illumina) in paired-end 150-bp mode. The transcript-level expression analysis was performed following the protocol proposed by Pertea et al. (2016) using HISAT2 (version 2.0.5) (Kim et al. 2015), StringTie (version: 1.3.6) (Pertea et al. 2015), and Ballgown (Frazee et al. 2015). The details are available in Supplemental Methods.

### RNA extraction and expression analysis using qRT-PCR

For *PpSVP* expression analysis, floral buds from “Nanshan Tian Tao” were sampled on October 15, November 15, and December 15 of 2016, and January 15, February 15, and March 15 of 2017. *PpLNK1* expression was measured in floral buds (blooming soon) from three global warming-sensitive accessions (“Wu Yue Xian”, “Nanshan Tian Tao”, and “Li He Pan Tao”) and three global warming-insensitive accessions (“Xinjiang Pan Tao”, “Wuhan 2”, and “Kashi 2”) in 2016 and 2017. For *PpAHP5*, the phloem (including cambium) was collected from two cultivars, “Hunchun Tao” (cold-resistant) and “Nanshan Tian Tao” (cold-sensitive), after 0, 6, 12, 24, 36, and 48 h of treatment under −28°C refrigeration and following 21°C incubation in water. For each sample, three biological replicates were used. Total RNA was extracted using an extraction kit (Aidlab), and first-strand cDNA was synthesized with 1 µg RNA using a FastQuant RT kit (with gDNase) (TIANGEN). Gene-specific primers were designed using the Primer-BLAST software (Ye et al. 2012). qRT-PCR was performed using a SYBR Green I master kit (Roche Diagnostics) with the LightCycler System (Roche LightCycler 480), following the manufacturer's protocol. Relative expression levels were calculated using the 2^−ΔΔCT^ method. The peach *ACT7* (*Prupe.6G163400*) gene was used as the reference.

### Global warming simulation experiment

The global warming simulation experiment was performed in 2016–2017. Three peach cultivars (Nanshan Tian Tao, Hu Jing Mi Lu, and Maravila), each with two clones, were used as plant materials. For each cultivar, ∼30 annual branches longer than 40 cm with floral buds were taken from each clone when the winter chill accumulation was ∼900 chilling hours (0°C–7.2°C, excluding 0°C). Branch cuttings were placed in water in a greenhouse at 25°C and with a 16 h/8 h photoperiod to simulate climate warming. The ratio of bud break was investigated daily, starting from the day that the branch cuttings were placed in the greenhouse. The floral buds, excluding the tegmentum, were collected weekly and frozen in liquid nitrogen. The sampled floral buds were used for qRT-PCR analyses following the protocol described above.

## Data access

All raw sequencing data generated in this study have been deposited in the NCBI BioProject database (https://www.ncbi.nlm.nih.gov/bioproject/) under accession number PRJNA388029 (SRX2915049, SRX2914970, and SRX2914939). The variation data generated in this study have been submitted to the European Variation Archive (EVA; https://www.ebi.ac.uk/eva/) under accession number PRJEB42015. All raw RNA-seq data have been deposited in the BioProject database under accession numbers PRJNA401307, PRJNA694007, PRJNA694195, and PRJNA694331.

## Competing interest statement

The authors declare no competing interests.

## Supplementary Material

Supplemental Material
