# Supplementary material for: Genomic analyses provide insights into peach local adaptation and responses to climate change
Source: Genome Res. 2021 Apr;31(4):592–606. doi: 10.1101/gr.261032.120 (PMC8015852; doi:10.1101/gr.261032.120)
Supplement: Supplemental Material [file supp_31_4_592__index.html]

Genomic analyses provide insights into peach local adaptation and responses to climate change — Genomic analyses provide insights into peach local adaptation and responses to climate change — Supplemental Material 

# Genomic analyses provide insights into peach local adaptation and responses to climate change

## Supplemental Material

- Supplemental\_Figures.docx
- Supplemental\_Tables.xlsx
- Supplemental\_Methods.docx
