# Supplementary material for: Genomic analyses provide insights into peach local adaptation and responses to climate change
Source: Genome Res. 2021 Apr;31(4):592–606. doi: 10.1101/gr.261032.120 (PMC8015852; doi:10.1101/gr.261032.120)
Supplement: Supplemental Material [file supp_gr.261032.120_Supplemental_Figures.docx]

**Genomic analyses provide insights into peach local adaptation and** **responses to climate change**

Yong Li,^1,2,3,8^ Ke Cao,^1,2,8^ Nan Li,^6^ Gengrui Zhu,^1,2^ Weichao Fang,^1,2^ Changwen Chen,^1^ Xinwei Wang,^1^ Jian Guo,^1^ Qi Wang,^1^ Tiyu Ding,^1^ Jiao Wang,^1^ Liping Guan,^1^ Junxiu Wang,^1^ Kuozhan Liu,^1^ Wenwu Guo,^3^ Pere Arús,^7^ Sanwen Huang,^6^ Zhangjun Fei,^4,5^ and Lirong Wang^1,2^

^1^Zhengzhou Fruit Research Institute, Chinese Academy of Agricultural Sciences, Zhengzhou 450009, China

^2^National Horticulture Germplasm Resources Center, Zhengzhou Fruit Research Institute, Chinese Academy of Agricultural Sciences, Zhengzhou 450009, China

^3^Key Laboratory of Horticultural Plant Biology (Ministry of Education), College of Horticulture & Forestry Sciences, Huazhong Agricultural University, Wuhan 430000, China

^4^Boyce Thompson Institute for Plant Research, Cornell University, Ithaca, New York 14853, USA

^5^U.S. Department of Agriculture-Agricultural Research Service, Robert W. Holley Center for Agriculture and Health, Ithaca, New York 14853, USA

^6^Agricultural Genome Institute at Shenzhen, Chinese Academy of Agricultural Sciences, Shenzhen 518000, China

^7^IRTA–Centre de Recerca en Agrigenòmica (CSIC-IRTA-UAB-UB), Barcelona 08193, Spain

^8^These authors contributed equally to this work.

Corresponding authors: Lirong Wang, wanglirong@caas.cn; Zhangjun Fei, zf25@cornell.edu


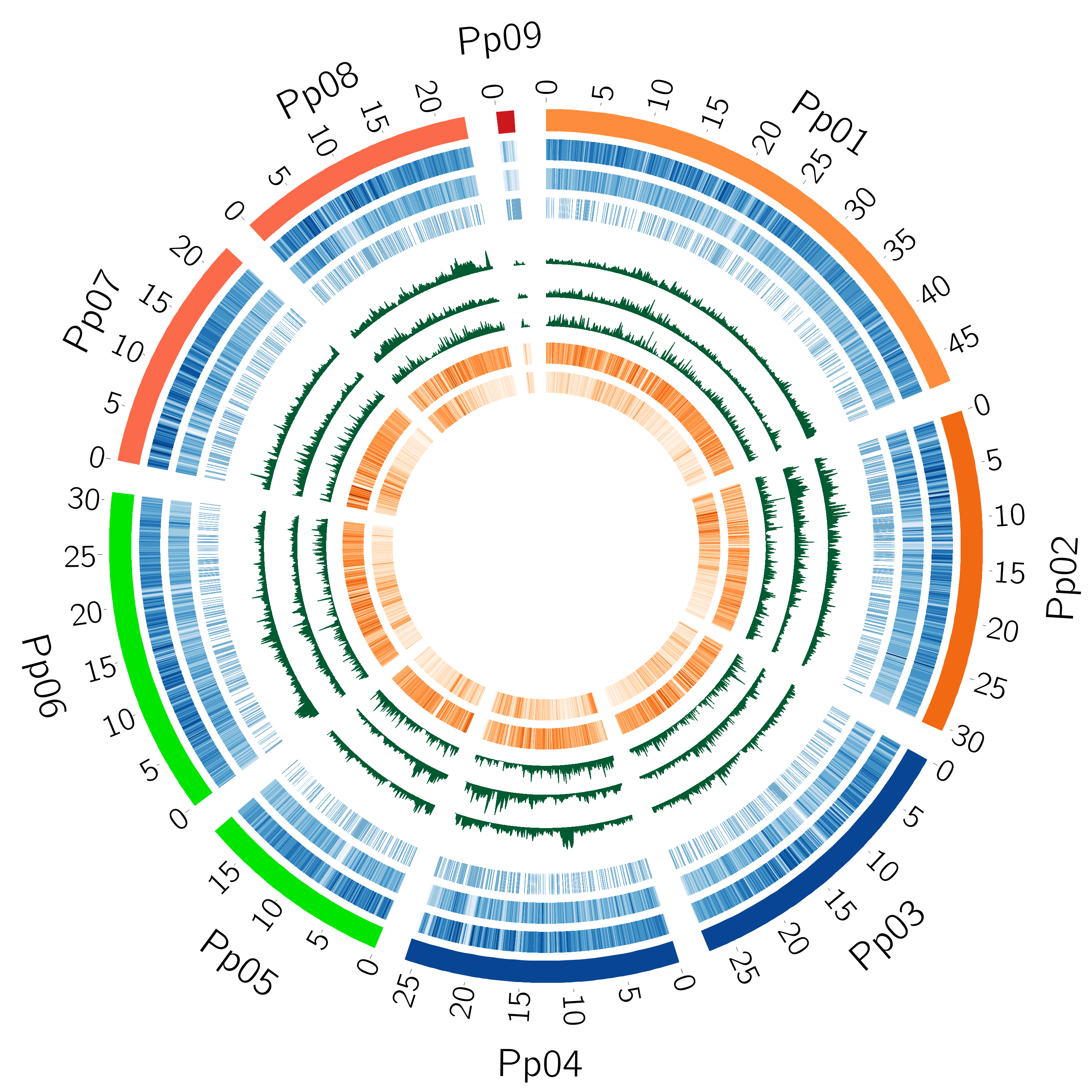


**A**

**B**

**Fig. S1** Genetic diversity and genetic relationship of 263 peach accessions. (**A**) Distribution of variants and genetic diversity of 263 peach accessions. The outer track represents eight chromosomes of the peach genome, and Pp09 indicates scaffolds that cannot be anchored on the eight chromosomes. The eight inner tracks depict (from the outside inward): SNP distribution, small indel (1-6 bp) distribution, large SV distribution, *Prunus mira* population nucleotide diversity (π), landrace population nucleotide diversity (π), large-effect SNP distribution, *P. mira* private SNPs, and landrace private SNPs. The circos plot in SVG format is available at figshare (https://figshare.com/articles/figure/Distribution_of_variants_and_genetic_diversity_of_263_peach_accessions_/13182422). (**B**) Neighbor-joining tree based on SNPs with minor allele frequency > 0.01. The layer rings indicate the group name of each clade. YG, Yun-gui Plateau. NW, Northwest China. NP, North Plain China. YT, Yangtze River Middle and Backward. NE, Northeast China. TB, Tibet plateau. ST, South China Sub-tropical. OR, ornamental accessions. WEM, wild relatives except *P. mira*.


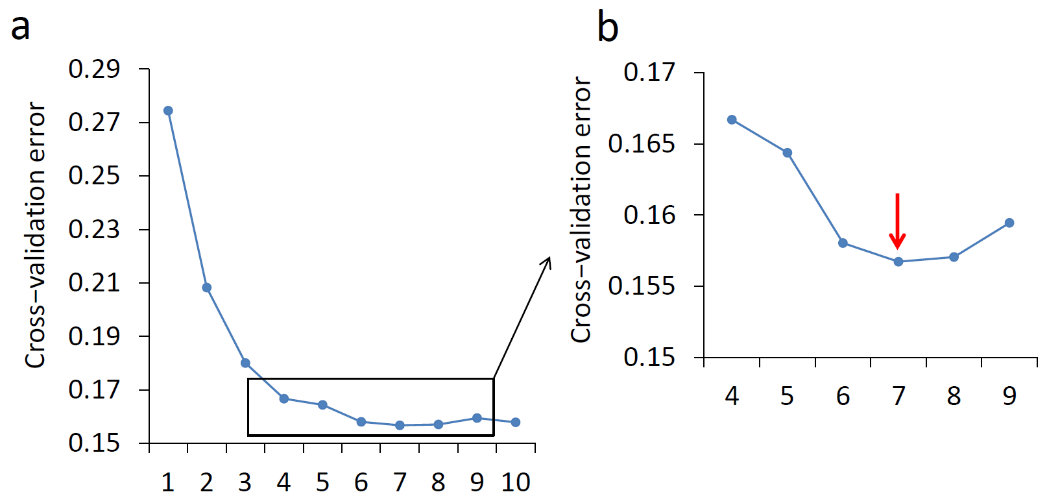


**A**

**B**

**E**


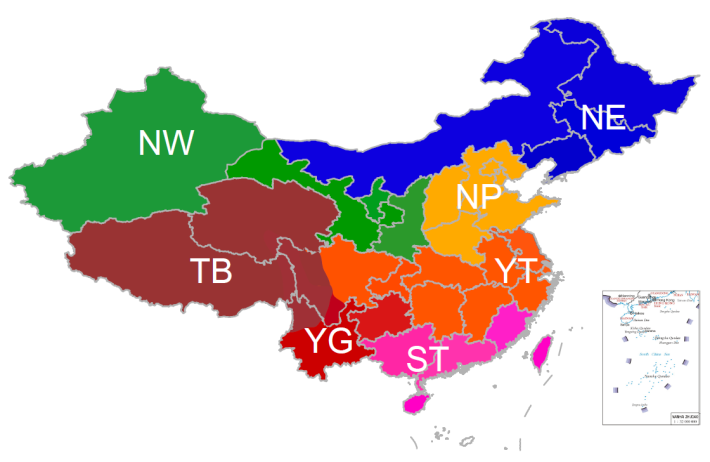


**C**


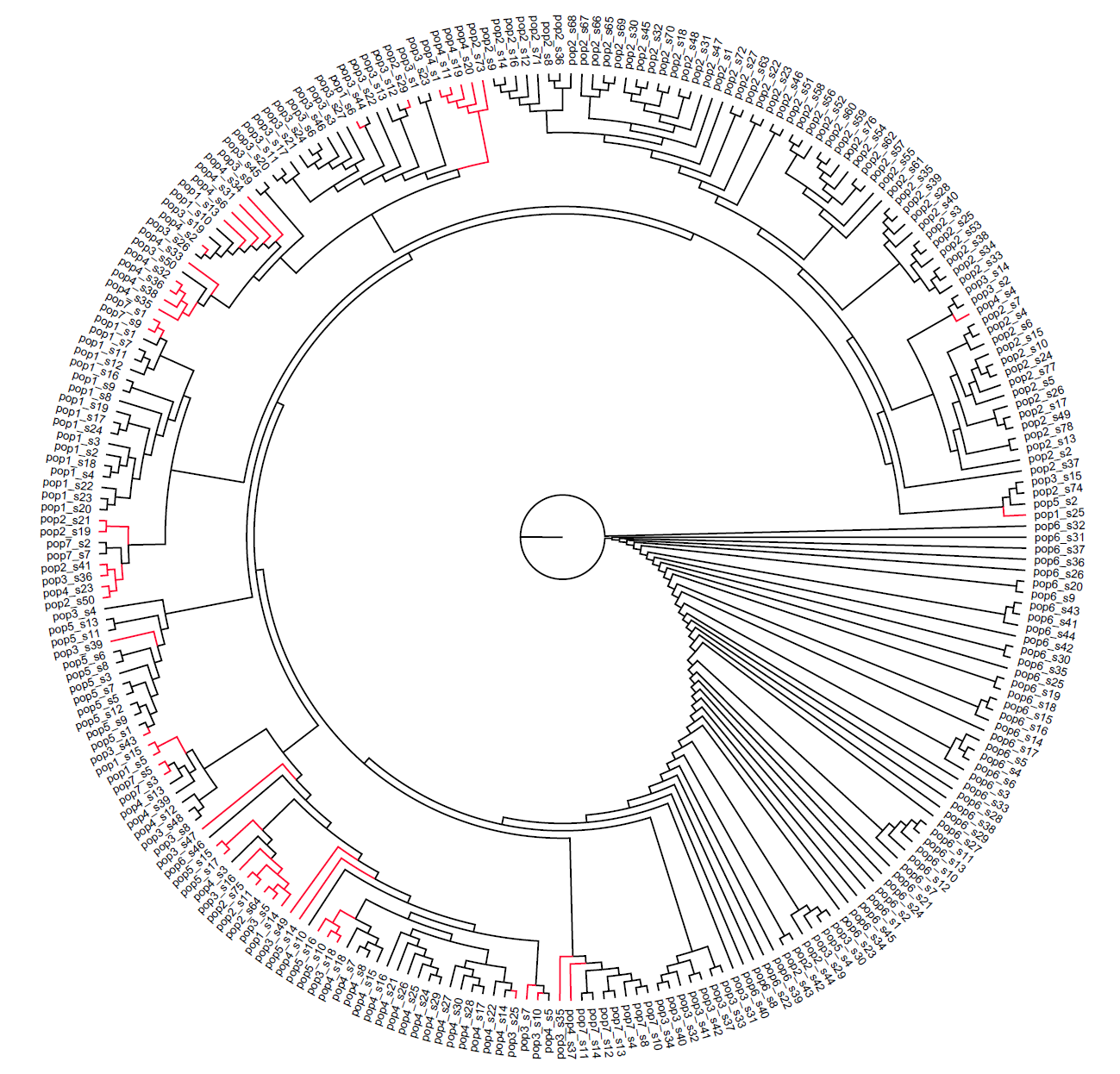


**D**

**F**

**G**

**H**


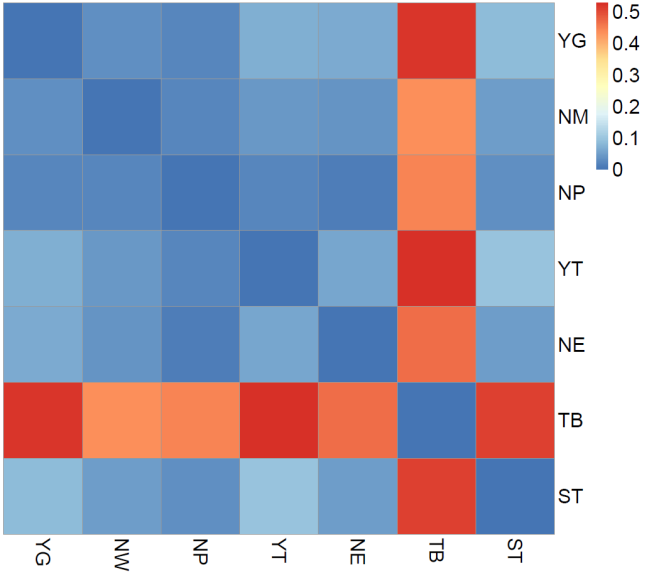


**Fig. S2** Genetic relationship and population structure of 263 peach accessions. (**A**) Cross-validation error estimation by increasing *K* from 1 to 10. (**B**) Zooming in the cross-validation error from *K*=4 to *K*=9. The cross-validation error showed a nadir at *K*=7 (red arrow), suggesting seven clusters as the optimal option. (**C**) Geographic regions of seven ecotypes of peach. Different ecotypes were represented by distinct colors. (**D**) Neighbor-joining tree of 263 peach accessions. Accessions showing discrepancies between geographical characterization and phylogenetic clustering are highlighted in red. The sample ID format is consistent with that in Table S1. “pop” and “id” were the abbreviation of “popu” and “sample”, respectively. (**E**) Population structure of peach accessions with *K* from 2 to 8. The y-axis indicates subgroup memberships, and the x-axis indicates the peach accessions. The orders and positions of these accessions on the x-axis are consistent with those for the neighbor-joining tree. ow indicates the wild relatives excluding *P. mira* and ornamental accessions. (**F**) PCA plot of the second and third components of 263 accessions. The inset shows PCA plot of the first and second components. TB, NW, and YG groups were marked using black circles. (**G**) Multidimensional scaling (MDS) plot for the pairwise *F*_ST_ matrix of the seven groups. The Euclidean distances between each pair of groups represent the corresponding *F*_ST_ values, which were calculated using R *stats* packages based on pairwise *F*_ST_. The nucleotide diversity for each group was calculated using VCFtools. The size of data points and the corresponding numbers indicate the nucleotide diversity π (10^-3^) of each group. (**H**) Heatmap of the pairwise *F*_ST_ values among the seven groups.

**A**

**B**

**C**

**D**

**E**

**F**

**G**

**Fig. S3** Over-represented Gene Ontology (GO) terms and Kyoto Encyclopedia of Genes and Genomes (KEGG) pathways in genes under selection in the seven peach groups. The *P* values for GO terms and KEGG pathway were corrected using Bonferroni and QVALUE adjustment, respectively. (**A**) YG group. (**B**) NW group. (**C**) NP group. (**D**) YT group. (**E**) NE group. (**F**) TB group. (**G**) ST group.


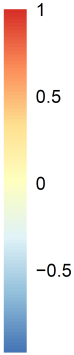

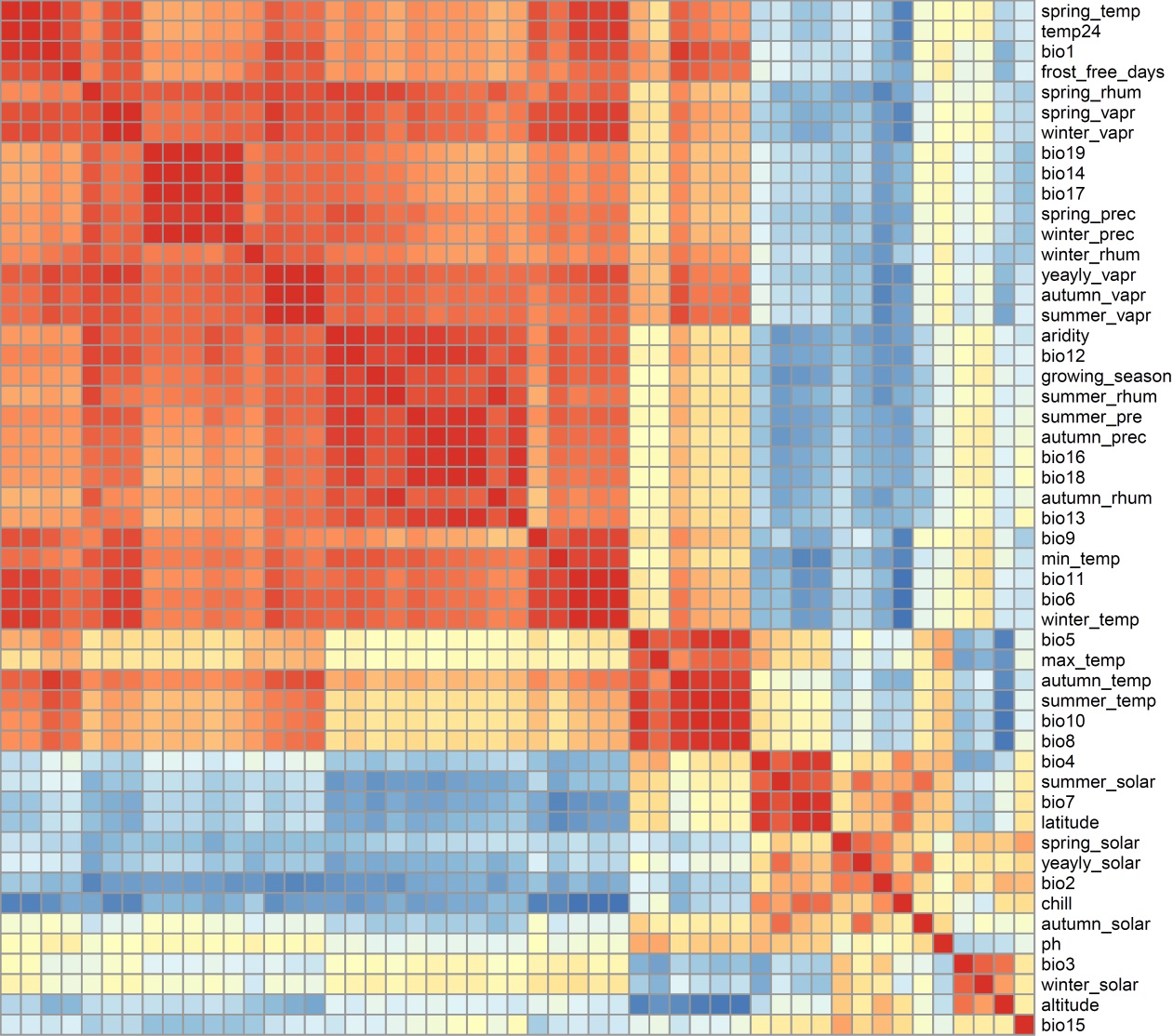

**Fig. S4** Pattern of correlations among different climate variables. Different colors indicate *Pearson* correlation coefficients.


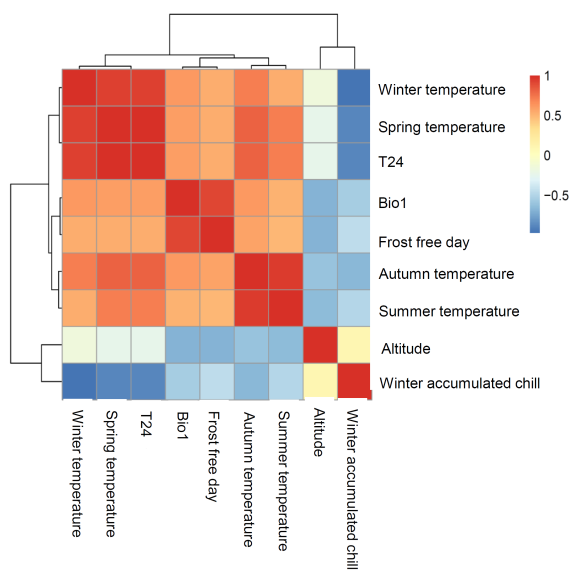


**D**

**B**

**C**

**A**

**a**

**c**

**…….**

**Fig.** **S5** Genome-wide associations for temperature- and precipitation-related traits. (**A**) Correlation of temperature-related climate variables. Different colors indicate Pearson correlation coefficients. (**B**) Circular Manhattan plots of the GWEAS for nine temperature-related environmental variables. The outer track represents eight chromosomes of peach genome. The inner order is (from the outside inward): annual mean temperature, mean temperature from Feb. to Apr., frost free days, winter accumulated chill, winter mean temperature, autumn mean temperature, summer mean temperature, spring mean temperature, and altitude. The Bonferroni significance threshold (1.09×10^-8^) is indicated by blue horizontal dashed lines. Strongly associated regions are pointed by blue arrows. The circos plot in SVG format is available at figshare (https://figshare.com/articles/figure/Circos_Manhattan_plot_for_GWEAS_in_peach_temperature/13168163). (**C**) Correlation of precipitation-related climate variables. Different colors indicate Pearson correlation coefficients. (**D**) Circular Manhattan plots of the GWEAS for seven precipitation-related environmental variables. The outer track represents eight chromosomes of peach genome. The order is (from the outside inward): length of the growing season, aridity index, annual mean precipitation, winter mean precipitation, autumn mean precipitation, summer mean precipitation, and spring mean precipitation. The Bonferroni significance threshold (1.09 × 10^-8^) is indicated by blue horizontal dashed lines. Strongly associated hotspot regions are pointed by blue arrows. Hotspots indicate association regions identified in at least five GWEAS. The circos plot in SVG format is available at figshare (https://figshare.com/articles/figure/Circos_Manhattan_plot_for_GWEAS_in_peach_precipitation/13168172).

**Fig. S6** Unique and shared genes between GWEAS and candidate selective regions (CSRs).

**
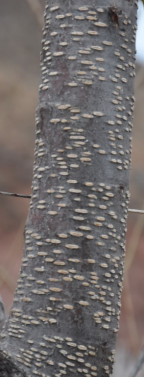

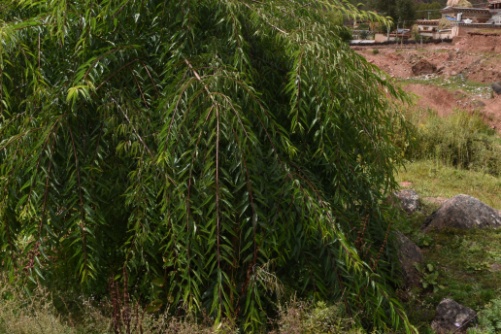
A**

**B**

**
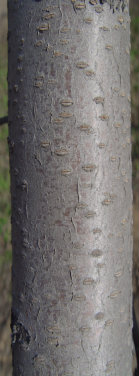
**

High altitude

Low altitude

**C**

**
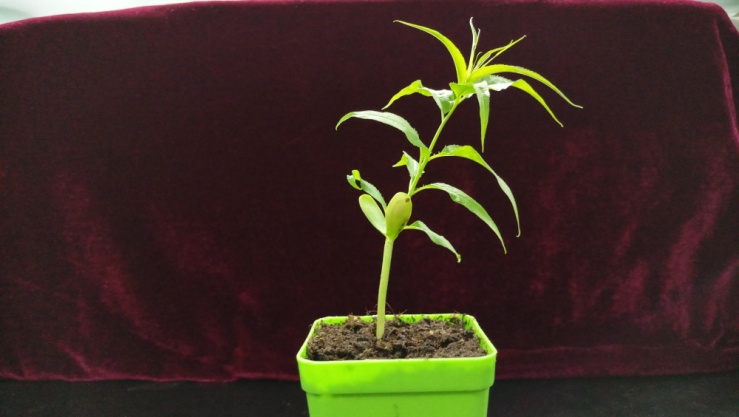

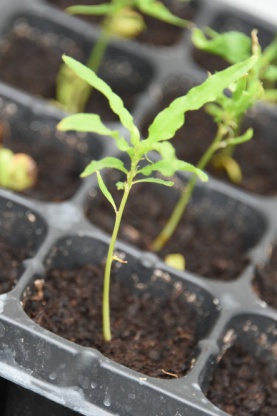
**

cotyledon

**D**

High altitude

Low altitude

**
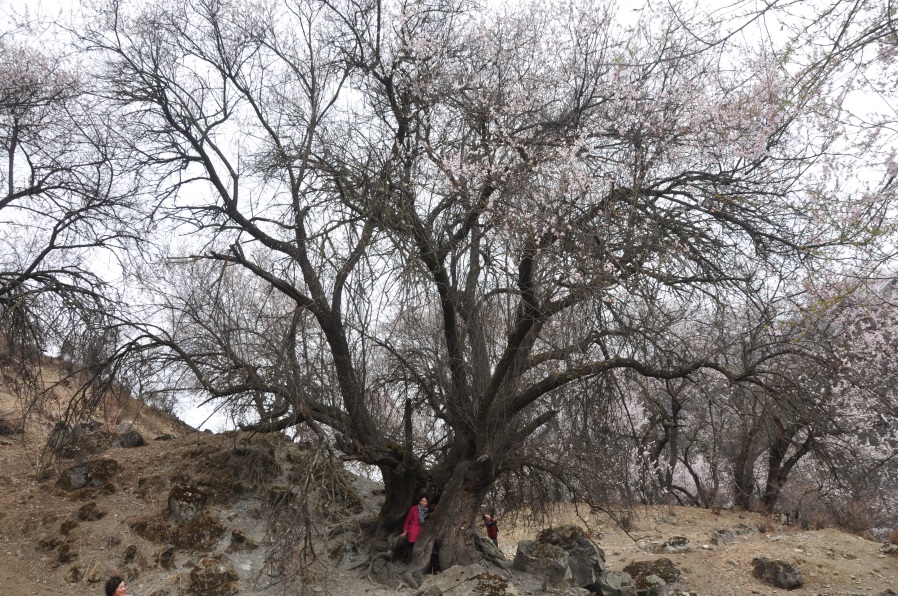
**

**Fig. S7** Comparison of the adaptive traits between accessions from low and high altitudes. (**A**) Red new shoot color under natural condition on the Tiber Plateau. (**B**) Branch colors and lenticel density of high and low altitude accessions. (**C**) Epigeal germination of accession in the TB group. The white arrow indicates cotyledon. (**D**) The long lifespan accession in the TB group (*Prunus mira*). The diameter was measured to be 2.1 m.

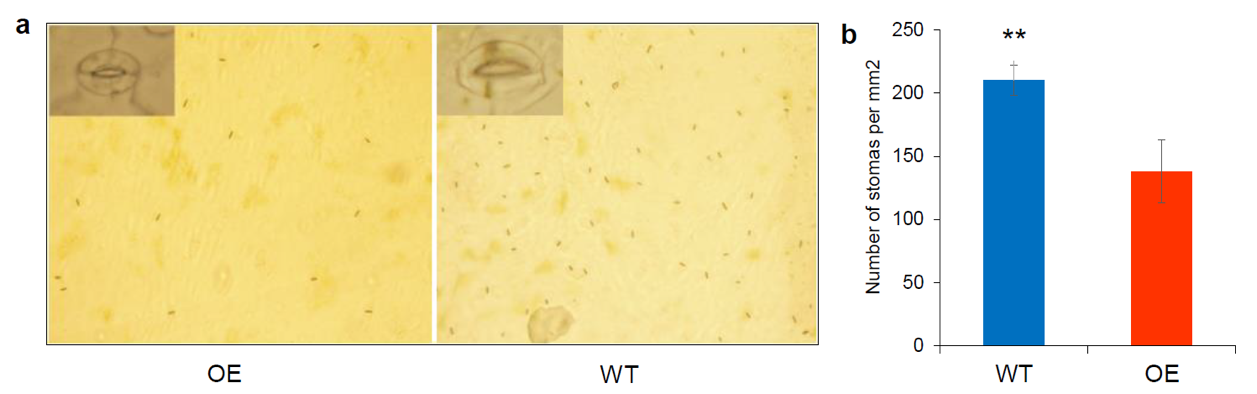
make

**A**

**B**

**Fig. S8** (**A**) Stoma in wild type and *PpEPF1* over-expression line. (**B**) Stoma density in wild type and *PpEPF1* overexpression lines. ** indicates *P* < 0.01.

**A**


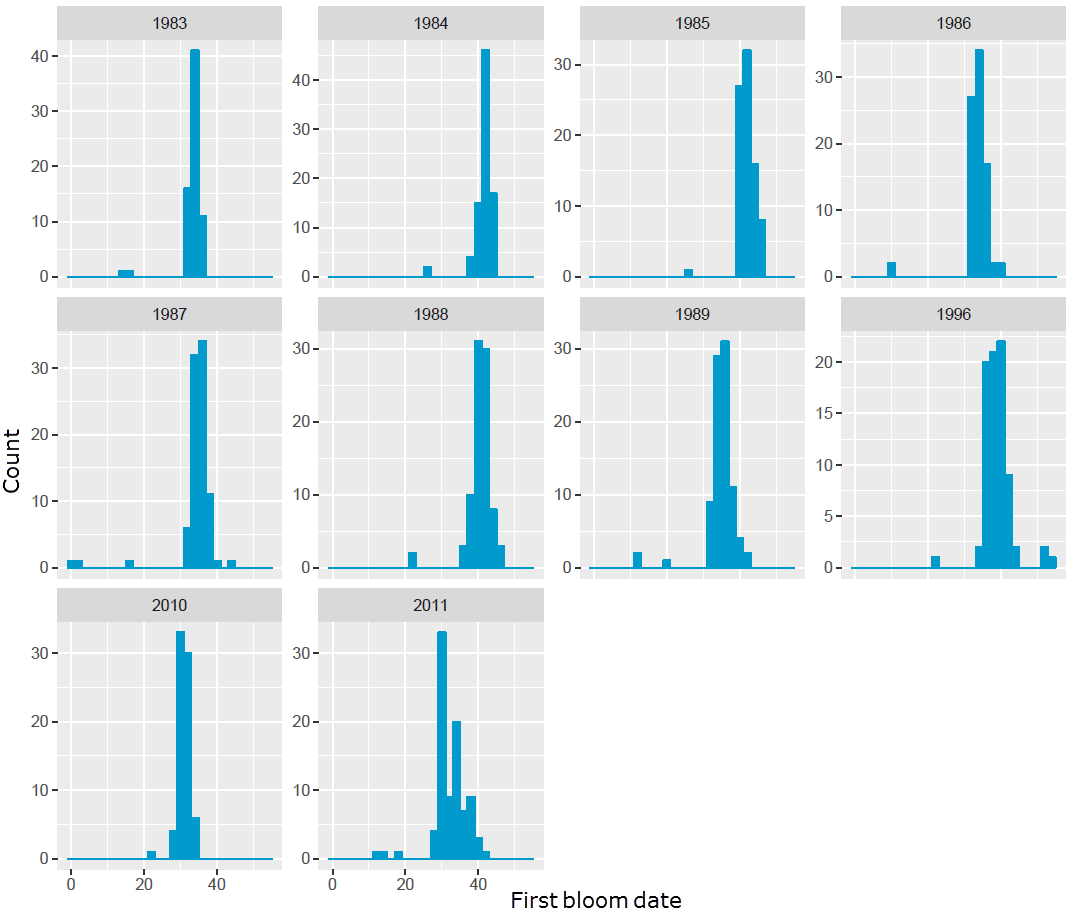


**B**

**Fig. S9** (**A**) Distribution of first bloom date for 89 peach accessions used in GWAS. (**B**) Distribution of advance of bloom date (ABD) for 89 peach accessions used in GWAS.
