# Supplementary material for: Genomic analyses provide insights into peach local adaptation and responses to climate change
Source: Genome Res. 2021 Apr;31(4):592–606. doi: 10.1101/gr.261032.120 (PMC8015852; doi:10.1101/gr.261032.120)
Supplement: Supplemental Material [file supp_gr.261032.120_Supplemental_Methods.docx]

**Genomic analyses provide insights into peach local adaptation and** **responses to climate change**

Yong Li,^1,2,3,8^ Ke Cao,^1,2,8^ Nan Li,^6^ Gengrui Zhu,^1,2^ Weichao Fang,^1,2^ Changwen Chen,^1^ Xinwei Wang,^1^ Jian Guo,^1^ Qi Wang,^1^ Tiyu Ding,^1^ Jiao Wang,^1^ Liping Guan,^1^ Junxiu Wang,^1^ Kuozhan Liu,^1^ Wenwu Guo,^3^ Pere Arús,^7^ Sanwen Huang,^6^ Zhangjun Fei,^4,5^ and Lirong Wang^1,2^

^1^Zhengzhou Fruit Research Institute, Chinese Academy of Agricultural Sciences, Zhengzhou 450009, China

^2^National Horticulture Germplasm Resources Center, Zhengzhou Fruit Research Institute, Chinese Academy of Agricultural Sciences, Zhengzhou 450009, China

^3^Key Laboratory of Horticultural Plant Biology (Ministry of Education), College of Horticulture & Forestry Sciences, Huazhong Agricultural University, Wuhan 430000, China

^4^Boyce Thompson Institute for Plant Research, Cornell University, Ithaca, New York 14853, USA

^5^U.S. Department of Agriculture-Agricultural Research Service, Robert W. Holley Center for Agriculture and Health, Ithaca, New York 14853, USA

^6^Agricultural Genome Institute at Shenzhen, Chinese Academy of Agricultural Sciences, Shenzhen 518000, China

^7^IRTA–Centre de Recerca en Agrigenòmica (CSIC-IRTA-UAB-UB), Barcelona 08193, Spain

^8^These authors contributed equally to this work.

Corresponding authors: Lirong Wang, wanglirong@caas.cn; Zhangjun Fei, zf25@cornell.edu

**Supplemental Methods**

**Plant materials and sequencing**

A total of 263 peach accessions were used in study, of which 218 were from the NPGRC (National Peach Germplasm Repository of China) and 45 *P. mira* accessions were sampled from the Tibet plateau. These accessions, collected from almost all the distribution regions of peach landraces and wild relatives in China, belonged to seven major ecotypes (Wang et al. 2001; Wang et al. 2012). These accessions included 45 of *P. mira* Koehne, 4 of *P. davidiana* (Carr.) Franch., 2 of *P. kansuensis* Rehd., a single *P. potaninii* Batal., 205 of *P. persica* L., and 6 of *P. ferganensis* Kost. et Riab (Supplemental Table S1), of which 260 have been reported in our previous study (Li et al. 2019) and 3 were new sequenced. Total genomic DNA of the three newly sequenced accessions was extracted from young leaves using the cetyltriethylammnonium bromide (CTAB) method (Murray and Thompson 1980). At least 4 μg of genomic DNA was used to construct pair-end sequencing libraries with insert sizes of approximately 500-bp following the manufacturer’s instructions (Illumina Inc.), which were sequenced on the Illumina HiSeq 2500 platform (Illumina, San Diego, USA) to generate 125-bp paired-end reads (Supplemental Table S1). Finally, a total of 1.3 Gb data for each accession were used for further analysis on average.

**Variation calling and annotation**

Pair-end reads from each accession were mapped to the peach ‘Lovell’ genome (release v2.0) using BWA (Li and Durbin 2009) (Version: 0.7.12) with the following parameters: bwa mem -t 4 -M -R. Read alignments were converted into the BAM format, sorted according mapping coordinates, and PCR duplicates removed using the Picard package (http://broadinstitute.github.io/picard/; Version: 1.136) with default parameters. The coverage and depth of sequence alignments were computed using the Genome Analysis Toolkit (GATK, version: 3.4-46) DepthOfCoverage program (McKenna et al. 2010).

To accurately identify SNPs, the low-quality alignments (a mapping quality score <20) were filtered using SAMtools (Li et al. 2009). SNP detection was performed using GATK HaplotypeCaller (Depristo et al. 2011). The detailed processes were as follows: (1) After filtering the low-quality alignments, the reads around the indels were realigned through two steps, including identifying regions where realignment was needed using the GATK RealignerTargetCreator package, and realigning the regions found in the first step GATK IndelRealigner package. Next, a realigned BAM file for each accession, which was used for SNP detection, was generated using GATK PrintReads packages. (2) SNPs were detected at a population level using the realigned BAM file with GATK HaplotypeCaller. To reduce the number of false positives, a high SNP confidence score was set with the following parameters: -stand_call_conf 30 -stand_emit_conf 40. (3) To ensure the quality of variant calling, a hard filter was applied for the raw SNPs with SNP quality > 40 and the number of supporting reads > 2, using GTAK VariantFiltration, with the following parameters: QUAL < 40, QD < 2.0, FS > 60.0, MQ < 40.0, MQRankSum < -12.5, ReadPosRankSum < -8.0, -cluster 3, -window 10.

The accuracy of SNPs was assessed using a Sequenom MassARRAY platform (Sequenom, San Diego, USA), following the manufacturer's protocol. A total of 18 randomly selected SNPs were investigated in 130 accessions.

Indel calling was performed using the same pipeline as the SNP calling since the GATK is capable of calling SNPs and indels simultaneously. To reduce the number of false positives, we also applied a harder filter for raw indels using GTAK VariantFiltration with the following parameters: QD < 2.0, FS > 200.0, ReadPosRankSum < -20.0. Inser­tions and deletions ≤6 bp were defined as the small indels.

SV calling was performed using the SpeedSeq (Chiang et al. 2015), DELLY (Tobias et al. 2012), and Manta (Chen et al. 2016) programs. For SpeedSeq calling, paired-end reads were mapped to the reference genome using the ‘align’ module in SpeedSeq and the following parameters: speedseq align -R -t 4. Three BAM files were generated, including a full, duplicate-marked, sorted BAM, a BAM file containing split reads, and a BAM file containing discordant read-pairs. SVs were identified using the ‘sv’ module in SpeedSeq, using the following settings: speedseq sv -o -x -t 25 -R -B -D -S -g -P. For DELLY calling, mapped pair-end reads in BAM format, generated by BWA-MEM (Li and Durbin 2009) after sorting and marking PCR duplicates, were used as input files. SVs were identified using the call module in DELLY with default parameters. SV files in VCF format for all of 263 samples were merged into a population level VCF file using BCFtools (Li et al. 2009). For SV calling with Manta, the same BAM files with DELLY were used to detect SVs, with default parameters. SV files for 263 accessions were then merged using SURVIVOR (Jeffares et al 2017) and genotyped using SVtyper (Chiang et al. 2015) with default parameters. Finally, SVs identified by at least two callers were designated as the final set of SVs.

SNP annotation was performed based on genomic locations and predicted coding effects, according to the peach genome annotation (release annotation v2.1), using the SnpEff (Cingolani et al. 2012) (Version: 4.1g). The final SNPs were categorized in exonic regions, intronic regions, splicing sites, 5' UTRs and 3' UTRs, upstream and downstream regions, and intergenic regions. SNPs in coding sequence were further grouped into synonymous SNPs (no amino acid changes) and nonsynonymous SNPs (amino acid changes). SNP effects were further divided into four types according to their impacts on gene function, including HIGH, MODERATE, LOW, and MODIFIER.

**Population genetics analysis**

To build a phylogenetic tree, we selected a subset of 3,429,878 SNPs with minor allele frequency (MAF) > 0.01 in all 263 accessions from the final SNP data set (4,611,842). A neighbor-joining tree was constructed using PHYLIP (Felsenstein 1989) (Version: 3.696) on the basis of the distance matrix with 1,000 bootstrap replicates. The software FigTree (http://tree.bio.ed.ac.uk/software/figtree/; version: 1.4.3) was used to visualize the neighbor-joining tree. The principal component analysis (PCA) was performed based on the same SNPs data set (3,429,878 SNPs with MAF > 0.01) using the smartpca program in the EIGENSFOT software (Version: 6.0.1) with default settings (Price et al. 2006). The first three eigenvectors were used to plot the data. The population structure was also investigated using the same SNP data set (3,429,878 SNPs with MAF>0.01) with the ADMIXTURE (Version: 1.1) software (Alexander et al. 2009). To determine the most likely cluster (*K*) number, cross-validation procedure in ADMIXTURE was ran for *K* values from 1 to 10. After determining the most likely K values (*K* =7), we ran ADMIXTURE to determine the group membership of each accession with 200 bootstrap replicates and *K* values from 2 to 8.

**Demographic history reconstruction**

The demographic history of seven groups was inferred using a hidden Markov model approach as implemented in Pairwise Sequentially Markovian Coalescence (PSMC) based on SNP distribution (Li and Durbin, 2011), with the following parameters: -N25 -t15 -r5 -b -p "4+25*2+4+6". The mean generation time was set at 7 years, assuming a mutation rate of 7.77×10^-9^ substitutions per site per generation (Xie et al. 2016).

**Collection of climate variables**

A total of 51 environmental variables were selected as being essential for peach growth and survival (Supplemental Table S6), representing extremes and seasonality of temperature and precipitation, altitude, latitude, relative air humidity, water vapor pressure, growing season lengths, and aridity. Of these, 39 datasets of climate variables were downloaded from the World Climate database (WorldClim) (http://www.worldclim.org; version: 1.4), with a resolution of 2.5 minutes, and climate variables for each accession were extracted using DIVA-GIS (http://www.diva-gis.org; version: 7.5) (Supplemental Table S6). Six climate variables were downloaded from the China Meteorological Data Center (CMDC) (http://data.cma.cn/en/?r=site/index) and climate variables for each accession were extracted using ArcGIS (http://www.arcgis.com; version: 10.3) (Supplemental Table S6). Four climate variables were downloaded from the Food and Agriculture Organization (FAO) GeoNetwork (http://www.fao.org/geonetwork/srv/en/main.home), with a resolution of 5 minutes or 10 minutes and climate variables for each accession were extracted using ArcGIS (Supplemental Table S7). Altitude and latitude for each accession were recorded using a GPS (Magellangps triton 300E; http://www.magellangps.com) when the accessions were collected.

**Genome-wide environmental association study (GWEAS)**

GWEAS was performed for 51 EVs using 211 landrace accessions based on 4,596,331 SNPs using the mixed linear model (MLM) with Efficient Mixed-Model Association eXpedited (EMMAX) software (Kang et al. 2010). The kinship matrix for 211 accessions was estimated using emmax-kin in EMMAX program. To minimize the number of false positives and increase statistical power, the kinship matrix and PCA (PC1, PC2, and PC3) were used as the random effect and fixed effect covariates, respectively. The genome-wide significance threshold was set as 0.05/total number of SNPs (log_10_(*P*) = -7.13) using the Bonferroni test. Due to the high degree of correlations among these EVs (Supplemental Fig. S4), we performed a PCA on 51 EVs using the “prcomp” function in R (Version: 3.3.4) to identify PCs that best summarized the range of environmental variation (R Core Team, 2018). The PCA result showed that the first environmental PC (PC1) explained > 60% of the total variance which can represent our overall environmental variable. The LFMM analyses was performed with PC1 of EVs and genome-wide SNPs using R package lfmm (Version: 1.3) with following parameters: -p 8 -K 3 -I 10000 (Frichot et al. 2013). The top 10 association signals for each EV were annotated on the peach genome using the pygtrait program (https://github.com/orangeSi/pygtrait).

**Phenotyping and GWAS for chilling requirement and cold hardiness**

For chilling requirement (CR), phenotyping analyses were performed in 2011 and 2012 as in Fan et al (2010) and Li et al (2019). A 0-7.2°C model was chosen to evaluate CR and the number of hours in this range (chilling hours; CHs) was counted, starting when the daily average air temperature dropped to below 7.2°C. Starting at 50 CHs, the branches of each accession were cut every 50 CHs until 1,300 CHs. For each accession, two clones were sampled, and three branches longer than 40 cm with floral buds were taken from each clone. Branch cuttings were placed in water in a greenhouse at 25°C and a 16 h/8 h photoperiod to force floral bud break. The frequency of floral bud break was evaluated after two weeks. The CR of an accession was defined as being sufficient at a specific sampling time if 50% of floral buds on the branch cuttings opened. GWAS for CR was performed GWAS was performed using the EMMAX program (Kang et al. 2010) with a set of SNPs with MAF > 0.05 and data missing rate < 0.2. The kinship matrix and PCA were used as the random effect and fixed effect covariates, respectively. The genome-wide significance threshold was set as 0.05/total number of SNPs (-log_10_(*P*) = 7.08) using the Bonferroni correction.

Cold hardiness was evaluated using a conductance-based semi-lethal temperature method described by Zhang et al (2019) in December-January of 2013-2014 on 143 accessions. Six annual branches longer than 20 cm were sampled for each accession. A total of six cold treatments were used: -10, -15, -20, -25, -30, and -35°C. Branch cuttings were incubated in freezer with the six treatments for 24 h. After cold treatments, the cuttings were placed at 0°C for 8 h. Branch cuttings were then cut into 2 mm segments. A total of 2 g of segments was used to measure the conductance, with three biological replicates. The initial conductance (C1) was measured after a 12 h steep in 10 ml water. The final conductance (C2) was measured after boiling the samples for 20 min and leaving them to cool to room temperature for a subsequent 2 h period. The relative conductance (RC) was calculated using following formula:

RC=(C1/C2) × 100

Finally, the semi-lethal temperature (LT50) was calculated using a logistic function based on RC. GWAS for cold hardiness was performed using the same method with CR.

**Scanning electron microscopy (SEM)**

Stomata were examined by SEM in young leaves of three accessions from the TB group and three accessions from the NP group planted in NPGRC (Zhengzhou, China), representing high-altitude and low-altitude accessions, respectively. Three replicates were sampled from each accession. Samples were fixed in 2.5% glutaraldehyde (pH = 7.4) for 24 h at 4**℃**. Subsequently, fixed samples were dehydrated using an ethanol series (30% ethanol, 20 min; 50% ethanol, 20 min; 70% ethanol, 20 min; 100% ethanol, 30 min (twice)). The dehydrated samples were then dried in a critical-point drying apparatus (Quorum K850; England). Dried samples were mounted on stubs and sputter-coated with gold (FEI; America) and observed under a scanning electron microscopy (SEM) (FEI Quanta 250; America).

**Yeast one-hybrid assay**

Yeast one-hybridization assay was performed using the Matchmaker^®^ Gold Yeast One-Hybrid System (Clontech, Palo Alto, CA, USA). The promoter sequence (2 kb upstream of the start codon) of the sucrose phosphate synthase, *PpSPS1* (*Prupe.1G483200*), was cloned into the pAbAi vector. Similarly, the full-length of ABA-responsive element binding 1, *PpAREB1* (*Prupe.1G434500*), was subcloned into the pGADT7 AD vector. The auto-activation and TF–protein interaction analyses were conducted according to manufacturer's protocol.

**Over-expression of candidate genes in A. thaliana**

The full-length open reading frames of three peach genes, *PpEPF1* (*Prupe.3G235800*) and *PpLNK1* (*Prupe.8G062200*), were amplified by PCR using cDNAs derived from young leaves of ‘Aba Guang He Tao’ and ‘Nanshan Tian Tao’, respectively. The PCR products were cloned into the pBI121 vector driven by the cauliflower mosaic virus (CaMV) 35S promoter. The resulting constructs were then transformed into *A. thaliana* Columbia ecotype using *Agrobacterium tumefaciens* GV3101, and positive transformants were selected with kanamycin. For *PpLnk1* over-expression, ten transgenic lines for each gene were used to evaluate the flowering time. For *PpEPF1* over-expression, the stomata size and density were observed under a light microscope (Olympus BX51, Tokyo, Japan) with a 400 × objective lens in ten lines.

**RNA-seq analysis**

For drought stress treatment, four-year-old potted peach seedlings from peach cultivar “Dong Xue Mi Tao” were used. Fruit flesh were taken at six drought stress treatment time points, including 6 hours, 12 hours, 24 hours, 3 days, 6 days, and 12 days. For expression profiles in different tissues, roots, leaves, fruit, flowers, phloem, and seeds were sampled from “Aba Guang He Tao” (high-altitude) and “B-4” (low-altitude). For the expression of *PpCCD4,* fruit fleshes were sampled from “Zao Huang Pan Tao” (yellow-fleshed) and “Zhong Tao Hong Yu” (white-fleshed) at 10, 50, and 90 days post bloom date (dpb). For the expression of *PpSPS1*, fruit fleshes were sampled from “Chinese Cling” at 20, 40, 60, 80, 100, 120 dpb. Three biological replicates were collected for each sample. The tissues were immediately frozen in liquid nitrogen and then ground to fine powder. Total RNA was extracted using a quick extraction kit (Aidlab, Beijing, China). First and second strand complementary DNA (cDNA) was synthesized using a cDNA Synthesis System kit (TOYOBO, Osaka, Japan), following the manufacturer’s protocol. Double-strand cDNAs were then purified and adapters were ligated to the short fragments. The constructed RNA-seq libraries were sequenced using the Illumina HiSeq 2000 platform (Illumina, San Diego, USA) in paired-end 150-bp mode. Low-quality reads were filtered from the raw reads using Trimmomatic (Version: 0.35) (Bolger et al. 2014). Cleaned reads were mapped to the peach reference genome using HISAT2 (Version 2.0.5) (Kim et al. 2015) with default parameters. Transcript abundances were calculated using StringTie (Version: 1.3.6) (Pertea et al. 2015). DEG analysis was carried out using the R package Ballgown (Frazee et al. 2015).

**References**

Alexander DH, Novembre J, Lange K. 2009. Fast model-based estimation of ancestry in unrelated individuals. *Genome Res* **19**: 1655-1664.

Bolger AM, Lohse M, Usadel B. 2014. Trimmomatic: a flexible trimmer for Illumina sequence data. *Bioinformatics* **30**: 2114-2120.

Chen X, Schulz-Trieglaff O, Shaw R, Barnes B, Schlesinger F, Källberg M, Cox AJ, Kruglyak S, Saunders CT. 2016. Manta: rapid detection of structural variants and indels for germline and cancer sequencing applications. *Bioinformatics* **32**: 1220-1222.

Chiang C, Layer RM, Faust GG, Lindberg MR, Rose DB, Garrison EP, Marth GT, Quinlan AR, Hall IM. 2015. SpeedSeq: ultra-fast personal genome analysis and interpretation. *Nat Methods* **12**: 966-968.

Cingolani P, Platts A, Wang L, Coon M, Nguyen T, Wang L, Land SJ, Lu X, Ruden DM. 2012. A program for annotating and predicting the effects of single nucleotide polymorphisms, SnpEff. *Fly* **6**: 2, 80-92.

Depristo MA, Banks E, Poplin R, Garimella KV, Maguire JR, Hartl C, Philippakis AA, del Angel G, Rivas MA, Hanna M, et al. 2011. A framework for variation discovery and genotyping using next-generation DNA sequencing data. *Nat Genet* **43**: 491-498.

Fan S, Bielenberg DG, Zhebentyayeva TN, Reighard GL, Okie WR, Holland D, Abbott AG. 2010. Mapping quantitative trait loci associated with chilling requirement, heat requirement and bloom date in peach (*Prunus persica*). *New Phytol* **185**: 917-930.

Felsenstein J. 1989. PHYLIP-phylogeny inference package (version 3.2). *Cladistics* **5**: 164-166.

Frazee AC, Pertea G, Jaffe AE, Langmead B, Salzberg SL, Leek JT. 2015. Ballgown bridges the gap between transcriptome assembly and expression analysis. *Nat Biotechnol* **33**: 243-246.

Frichot É, Schoville SD, Bouchard G, François O. 2013. Testing for associations between loci and environmental gradients using latent factor mixed models. *Mol Biol Evol* **30**: 1687-1699.

Jeffares DC, Jolly C, Hoti M, Speed D, Shaw L, Rallis C, Balloux F, Dessimoz C, Bähler J, Sedlazeck FJ. 2017. Transient structural variations have strong effects on quantitative traits and reproductive isolation in fission yeast. *Nat Commun* **8**: 14061.

Kang HM, Sul JH, Service SK, Zaitlen NA, Kong S, Freimer NB, Sabatti C, Eskin E. 2010. Variance component model to account for sample structure in genome-wide association studies. *Nat Genet***42**: 348-354.

Kim D, Langmead B, Salzberg SL. 2015. HISAT: a fast spliced aligner with low memory requirements. *Nat Methods* **12**: 357-360.

Li H, Durbin R. 2009. Fast and accurate short read alignment with Burrows-Wheeler transform. *Bioinformatics* **25**: 1754-1760.

Li H, Handsaker B, Wysoker A, Fennell T, Ruan J, Homer N, Marth G, Abecasis G, Durbin R, 1000 Genome Project Data Processing Subgroup. 2009. The sequence alignment/map format and SAMtools. *Bioinformatics* **25**: 2078-2079.

Li H, Durbin R. 2011. Inference of human population history from individual whole-genome sequences. *Nature* **475**: 493-496.

Li Y, Cao K, Zhu G, Fang W, Chen C, Wang X, Zhao P, Guo J, Ding T, Guan L, et al. 2019. Genomic analyses of an extensive collection of wild and cultivated accessions provide new insights into peach breeding history. *Genome Biol* **20**: 36. doi:10.1186/s13059-019-1648-9

Murray M, Thompson WF. 1980. Rapid isolation of high molecular weight plant DNA. *Nucleic Acids Res* **8**: 4321-4326.

McKenna A, Hanna M, Banks E, Sivachenko A, Cibulskis K, Kernytsky A, Garimella K, Altshuler D, Gabriel S, Daly M, et al. 2010. The Genome Analysis Toolkit: a MapReduce framework for analyzing next-generation DNA sequencing data. *Genome Res* **20**: 1297-1303.

Pertea ML, Pertea GM, Antonescu CM, Chang TC, Mendell JT, Salzberg SL. 2015. StringTie enables improved reconstruction of a transcriptome from RNA-seq reads. *Nat Biotechnol***33**: 290-295.

Price AL, Patterson NJ, Plenge RM, Weinblatt ME, Shadick NA, Reich D. 2006. Principal components analysis corrects for stratification in genome-wide association studies. *Nat Genet* **38**: 904-909.

R Core Team. 2018. *R: A Language and Environment for Statistical Computing*. R Foundation for Statistical Computing, Vienna. https://www.R-project.org/.

Tobias R, Zichner T, Schlattl A, Stütz AM, Benes V, Korbel JO. 2012. Delly: structural variant discovery by integrated paired-end and split-read analysis. *Bioinformatics* **28**: i333-i339.

Xie Z, Wang L, Wang L, Wang Z, Lu Z, Tian D, Yang S, Hurst LD. 2016. Mutation rate analysis via parent–progeny sequencing of the perennial peach. I. A low rate in woody perennials and a higher mutagenicity in hybrids. *Proc Biol Sci* **283**: 20161016. doi:10.1098/rspb.2016.1016

Wang Z, Zhuang E. 2001. Geographical distribution and cultivation regions of peach in China. *Fruit trees of China, peach volume*. 49-51. China Forestry Press, Beijing, China.

Wang L, Zhu GR, Fang WC*.* 2012. Genetic diversity of Chinese peach landraces and elite landrace cultivars. *Peach genetic resource in China*. 201-212. China Agriculture Press, Beijing, China.

Zhang J, Wu X, Niu R, Liu Y, Liu N, Xu W, Wang Y. 2012. Cold-resistance evaluation in 25 wild grape species. *Vitis* **51**: 153-160.
